# A review to determine regulatorily and reimbursement successes of studies conducted using data from Canadian patient support programs based on the real-world evidence guidelines published by Canadian drug agency and health Canada

**DOI:** 10.3389/jpps.2025.14587

**Published:** 2025-08-08

**Authors:** Catherine Y. Lau

**Affiliations:** Consultant, Toronto, ON, Canada

**Keywords:** regulatory, patient-support-program (PSP), Canada’s-drug agency CDA-AMC, RWE-checklist, health Canada

## Abstract

**Introduction:**

Patient Support Programs (PSPs) are growing globally to support early reimbursement, disease and medication dosing management. In Canada, the lack of public health support has promoted the rapid expansion of company-supported disease-specific or drug-product-specific PSPs. Data collected from these programs generate unique Canadian data serving as a valuable source of real-world data (RWD), generally adopted in EU and US as a source of evidence generation. This review evaluates the suitability of PSP data for regulatory or reimbursement submissions, based on recently published Real World Evidence guidelines by the Canadian Drug Agency (CDA-AMC).

**Methods:**

Peer-reviewed publications evaluating patients with chronic diseases enrolled in a PSP from 1 January 2020, to 31 March 2025, were selected for review. The checklist in the CDA-AMC RWE Guideline was used to measure the quality and suitability of the PSP data.

**Results:**

Nine studies were reviewed against the checklist. Based on the criteria required to inform decision-making, most studies failed to meet key criteria for regulatory submissions. One recently published study, “Therapeutic Drug Monitoring of Infliximab” met most regulatory and reimbursement submission requirements.

**Conclusion:**

Data quality validation, data source transparency, validated methodology to manage study bias, measured or unmeasured confounders, and robust outcome analysis, including sensitivity and quantitative bias analysis, are essential to ensure PSP data analysis results in successful decision-making.

## Introduction

Real world evidence (RWE) in medicine is evidence on the use, safety, effectiveness, and cost of health technologies, which is observational data obtained outside the context of randomized controlled trials (RCTs) and generated during routine clinical practice. RWE is generated by analyzing data obtained from patient registries, medical records, or in some cases hybrid trials, pragmatic trials, and late-phase trials [1, 2]. As part of an effort to accelerate medical product development and bring innovations faster and more efficiently to patients who need them, the 21st Century Cures Act was signed into law on December 13, 2016, with the US FDA issuing its “Framework for FDA’s Real-World Evidence Program” in December 2018 [3]. Since then, the FDA has updated the guidance multiple times to assist with using RWE to approve a new indication for a drug [4, 5]. Health Canada followed suit and, on April 16, 2019, published the document “Optimizing the Use of Real-World Evidence to Inform Regulatory Decision-Making,” acknowledging that the use of RWE in regulatory decisions is increasing globally in the assessment of drug safety, efficacy, and effectiveness [6]. However, upon review of regulatory reports supporting approvals, Health Canada’s use of RWE in regulatory decision-making was considerably lower than that of European Medicine (EU) and US FDA [7]. Recently, on April 2023, the Canadian Drug Agency (CDA-AMC) and Health Canada jointly published a submission-ready RWE guidance outlining a step-by-step path to prepare quality documents for submission [8]. The Canadian guidance document, more so than other regulatory guidance, stresses the importance of “Transparency” in every step of the submission preparations. In the United States, the availability of sophisticated databases from electronic health and medical records provides rich platforms for generating RWE based on routine clinical practice outside clinical trials [4, 5]. While the utility of provincial, institutional datasets and registries can provide valuable data sources for RWE, the absence of a unified, pan-Canadian database underscores the importance of PSP capturing nation-wide patient data. Canada and Europe struggle to access large national databases for real-world data to generate RWE. The lack of robust common databases and the strength of patient privacy laws appear to be the most prominent obstacles [9]. In Canada, the arrival of specialty drugs in the past decade has promoted the growth of company-supported, nationwide PSPs. The goal of PSP is to help patients navigate the complex path of the drug reimbursement environment, assist with dosing administrations (including infusions/injections and training), manage side effects, and provide general patient support [10]. Most programs collect patient demographic data, dosing information at initiation and subsequent changes, and critical laboratory data essential to patient management and disease activities. As drug utilization is a key measurement yardstick, most programs collect individual patients’ data from the start to the end of treatment, providing essential data for drug adherence and persistence [11]. Moreover, Patient Support Programs (PSPs) offer valuable insights into the early effectiveness and safety profiles of drugs. These programs often provide critical data points, such as optimal treatment duration and potential signals for early withdrawal. This information not only aids in refining treatment protocols but also supports healthcare providers in making evidence-informed decisions that enhance patient outcomes.

With the lack of national and, in some cases, even provincial databases, will pan-Canadian PSP databases be able to fill the gap for the generation of RWE? Several recent publications advocate using PSP data as a source of RWD and assess whether the RWE generated meets regulatory/reimbursement requirements [10,11]. This publication reviewed peer-reviewed studies from 1 January 2020, to 28 February 2025, based on data from PSPs enrolling patients with chronic diseases. The recently published CDA RWE guidance was used to evaluate the appropriateness of the data source and subsequent analysis for submission purposes.

## Methods

### Selection of published studies using PSP data

This review focused on peer-reviewed articles published between 1 January 2020, and 28 February 2025. Only company-sponsored PSPs for chronic diseases (non-oncology) were selected to ensure adequate size of the program and sufficiently long follow-up time. As most RWE guidelines were published initially around 2016–2019, peer-reviewed articles published from January 2020 onwards were selected to ensure authors would be familiar with various guidelines published by regulatory or reimbursement agencies. Several search engines were used, including Google, Google Scholar, Pubmed, and Microsoft Academia, to search for peer-reviewed articles. In addition, the websites of Disease- Associations in Canada which list company-sponsored PSPs were reviewed, and the drugs listed were used to search for articles on Google Scholar. Links to Canadian disease associations with PSPs and medications included in PSPs are shown in Supplementary Table S1. A Google Scholar search was performed on all medicines listed in Supplementary Table S1 to capture PSP publications from Jan 1, 2020, to Feb 28, 2025. Nine publications were identified for further analysis (Table 1) [12–20]. The selection was confirmed with another comprehensive database of the Canadian PSP websites listed up to August 2023 [21].

**TABLE 1:** Studies Selected for Evaluations using CDA RWE Submission Guidelines.

Name of the drug (date of publication)	PSP program	Disease indicated	Size of patient population (N)	Effectiveness measured
^a^Infliximab (Remicade) [12] Feb 2025	BioAdvance	Inflammatory Bowel Diseases	13203	Persistence after Therapeutic Drug Monitoring and dose optimization
^a^Videolizumab (Entyvio) [13] March 2024	OnePath	Inflammatory Bowel Diseases	436	Relation between drug concentration and symptoms
^a^Mepolizuman (Nucala) [14] Feb 2024	MYNUCALA	Asthma	275	Treatment outcomes compared between beginning to end of treatment
^a^Brodalumab (Siliq) [14] April 2023	SILIQ patient support program	Plaque Psoriasis	864	Clinical symptoms improvement and persistence
Ustekinumab (Stelara) [16] July 2023	BioAdvance	Inflammatory Bowel Disease	8724	Treatment Persistence
Ixekizumab (Taltz) [17] July 2023	LillyPlus Support Services	Plaque Psoriasis	1891	Treatment persistence
Tofacitinib (Xeljanz) [18] Feb 2023	eXel	Rheumatoid Arthritis	4276	Treatment pattern and persistence
Dimenthyl Fumarate (Tecfidera) [19], April 2022	Biogen ONE	Multiple Sclerosis	12608	Treatment adherence and persistence
Erenumab (Aimovig) [20] August 2021	Go Program	Migraine	14,282	Treatment persistence

^a^ Studies grouped under Interventional (outcome based).

### Analysis of the peer-reviewed articles using PSP data

#### CDA RWE checklist

The nine selected articles and their Supplementary Material were reviewed for regulatory or reimbursement assessment, using the CDA RWE guidance checklist for suitability (Supplementary Tables S2–S10). The checklist has 12 sections: 1. Study design and research questions, 2. Setting and content, 3. Data specifications, access, cleaning methods and linkage, 4. Data sources, data dictionary and variables, 5. Participants, 6. Exposure definitions and comparators, 7. Outcomes, 8. Bias, confounding, effect modifiers or subgroup effects, 9. Statistical methods, 10. Study findings, 11. Interpretation and generalizability, 12. Limitations. Each item has 2–12 points for consideration. All publications were graded individually against each point with the page number(s) (section numbers if available) from the publication entered under the column “Reported on page” if reported. Under the column “If not reported or applicable, justify why,” the entries were NR (not reported) or NA (not applicable) (Supplementary Tables S2–S10).

Three sections have sub-bullets, 2.1 (2), 2.4 (4), 5.6 (3) and 9.3 (7). Sections 2.1, 2.4, and 5.6 could be grouped with one score, whereas, for section 9.3 on statistical methods, the 7 points must be addressed separately as studies vary in statistical stringency. The total score for the checklist is 103. Meanwhile, the score for NA should stay relatively consistent with the articles reviewed as the settings are based on company/drug-oriented PSPs. The lower score on NR represents better compliance with the RWE checklist. Each article was scored separately and unbiasedly (Tables 2, 4).

**TABLE 2:** Interventional studies selected for evaluations.

CDA guidelines sections (items)	Infliximab in IBD^a^		Vedolizumab in IBD^a^		Mepolizumab in asthma		Brodalumab in PsA	
Reporting Scores	NR	NA	NR	NA	NR	NA	NR	NA
1. Study design and research questions [11]	0	0	1	0	0	0	4	0
2. Setting and content [4]	0	1	1	1	0	1	1	1
3. Data specifications, access, cleaning methods and linkage [10]	0	8	1	8	1	1#	2	8
4. Data Sources, data dictionary and variables [12]	2	2	2	2	1	0#	5	2
5. Participants [3]	1	1	3	1	1	1	3	1
6. Exposure definitions and comparators [8]	0	5	0	5	0	5	0	5
7. Outcomes [7]	1	1	2	1	0	1	1	1
8. Bias, confounding, and effect modifiers or subgroup effects [11]	2	1	11	1	11	0	11	0
9. Statistical Methods [5]	1	1	3	1	3	1	3	1
10. Study findings [8]	2	0	3	0	3	0	6	0
11. Interpretation and generalizability [7]	1	0	2	0	2	0	2	0
12. Limitations [2]	0	0	0	0	0	0	0	0
Total	10	20	29	20	22	10#	38	19

NR, Not Reported; NA, Not Applicable.

^a^ With Supplementary Data published. # Sections 3 and 4 applied to this study as multiple data sources and vendor databases were used.

### Articles selected for analysis

Following FDA guidance [3, 4], the nine articles can be grouped under the Interventional and non-interventional categories (Table 1).

Interventional (Outcome based): According to the study protocol, study participants are assigned to one or more interventions to evaluate their value for studying outcomes.

1. Infliximab (Remicade) in Inflammatory Bowel Disease (IBD): Patients’ serum levels were measured after induction dosing to determine whether a dose increase was required for longer persistence [12] (Supplementary Table S2).
2. Vedolizumab (Entyvio) in IBD: Patients’ serum levels were measured 4–6 weeks after dose induction to evaluate correlation to study effectiveness [13] (Supplementary Table S3).
3. Brodalumab (Siliq) in Plaque Psoriasis: Efficacy outcomes were compared between baseline and end of the study with the drug being the intervention [15] (Supplementary Table S4).
4. Mepolizuman (Nucala) for the treatment of severe eosinophilic asthma symptoms with treatment as the intervention [14] (Supplementary Table S5).

Noninterventional: Participants are identified as belonging to a study group according to the drug or drugs received following routine medical practice and subsequent study outcomes evaluated.

1. Ustekinumab (Stelara) in IBD: A cohort study measuring patient persistence to the study drug [16] (Supplementary Table S6).
2. Ixekizumab (Taltz) in Plaque Psoriasis: A cohort study to assess treatment persistence [17] (Supplementary Table S7).
3. Tofacitinib in Rheumatoid Arthritis (RA): A cohort study to study treatment pattern [18] (Supplementary Table S8).
4. Dimethyl Fumarate (Tecfidera) in Multiple Sclerosis (MS): A cohort study to measure adherence and treatment persistence [19] (Supplementary Table S9).
5. Erenumab (Aimovig) A cohort study to measure persistence in patients with chronic and episodic migraine [20] (Supplementary Table S10).

#### Analysis performed to evaluate submission suitability

The two groups of published articles (interventional and noninterventional) were compared separately based on total scores from the CDA-AMC RWE Checklist. Each article was semi-quantitatively analyzed with the checklist score and descriptively commented on using additional 5 critical factors which summarize the 12 items on the CDA-AMC checklist: 1, Transparency in Data Collection and Reporting (items 1, 3,12); 2, RWD data sources and data validations (item 4); 3, study settings and Study population (items 2, 5, 6); 4, Study Monitoring; and (items 7,10) 5, Robust statistical analysis (items 8, 9,11).

#### Additional analysis conducted on interventional studies

The four interventional studies were further analyzed to determine suitability for submission purposes. Issues commonly arise in observational research that lead to erroneous interpretations and conclusions, and these issues were examined for the four studies according to “Common Issues with Biostatistics” for cohort studies [21]. These include the adequacy of study design, data quality handling, misunderstanding of confounders, and bias from group membership being attributed to future exposure in a retrospective study, such as immortal time and selection bias (Table 3).

**TABLE 3:** Evaluations of the design and statistical issues with interventional studies using PSP data [21].

	Subgroups prospectively designed to avoid bias	Adequate handling of databases, patient disposition	Applying sensitivity analysis	Adjusting for confounders and mediators	Account for immortal time bias	Account for selection bias
Infliximab in IBD	No	yes	yes	yes	yes	yes
Vedolizumab in IBD	No	no	yes	no	no	no
Mepolizumab in Asthma	No	yes	no	no	no	no
Brodalumab in Psoriasis	no	no	no	no	no	no

## Results

The group under interventional studies had the highest potential for regulatory or reimbursement submissions as they evaluated interventions that could support claims of efficacy or cost-effectiveness [4]. In addition to assessment using the CDA-AMC checklist, the validity of the studies was further examined under “Common Mistakes in Biostatistics” for cohort studies. The non-interventional studies focused on longitudinal follow-up of patients remaining on the drug or the program, and these patient persistence data are generally not candidates for regulatory submissions.

Table 1 lists the nine studies selected for evaluation. The first four studies were grouped under interventional and the last five as noninterventional.

### Interventional studies

Table 2 lists CDA-AMC checklist results for the four interventional studies based on PSP databases. The not applicable (NA) scores were the same for the infliximab, vedolizumab, and one point higher for the brodalumab study, as no sensitivity analysis was conducted (Section 8, point 8). The Mepolizumab study merged the PSP database with the ICES (Institute for Clinical Sciences) database, and many items in sections 3 and 4 apply to the study, resulting in an NA score of 10. The Mepolizumab study met all but one of the points for consideration of sections 3 and 4 so the NR score was not impacted compared to the other three products. The infliximab study, scored 10 for not reported (NR) and was the lowest among the four studies, followed by the mepolizumab study 22, the vedolizumab study 29, and the brodalumab study 38. In section 1, the brodalumab study had a higher NR of 4 due to a lack of study protocol, patient disposition chart, study committee, and formal ethics approval. All four studies scored well against Sections 2 and 3 with few NRs. The higher score of Brodalumab than the other three in Section 4 was mainly due to a lack of data transparency. The most significant differences in NR scores between the infliximab study and the other three studies were in Section 8, where selection bias, confounders, effect modifiers, or subgroup effects were discussed. The infliximab study adopted the Cox proportional hazards model with the intervention (therapeutic drug monitoring) as the time covariant to avoid immortal time bias, which was not used by other studies. Quantitative models (Quantitative Bias Analysis) were developed to evaluate the potential impact of measured and unmeasured confounders. None of the other studies mentioned bias or measured or unmeasured confounders. Each patient’s outcome in a specific subset in the infliximab study was artificially increased or decreased to determine the magnitude of adjustment required to contradict the observed result on comparisons between patient subsets. Such validation was not conducted in the other studies. Only two studies, the infliximab and the vedolizumab mentioned protocols were designed *a priori* for the analysis, and only two studies, infliximab and mepolzumab, provided patient disposition tables. The brodalumab study scored worse than the others in Section 10 regarding Study Findings as the study did not control for variables during the follow-up period, did not account for patient attrition during follow-up, comparing outcomes of patients that remained in the program to all patients at program initiation was selection bias, leading to erroneous interpretations.

The infliximab study is accompanied by a 200-page Supplementary Material, transparently displaying all the tables used for quantitative bias analysis, subgroup interactions, and sensitivity analysis. The study also completed a STROBE (Strengthening the Reporting of Observational Studies in Epidemiology) checklist, like the CDA-AMC RWE checklist, with satisfactory results. When evaluated against a recent publication on “Common mistakes in Biostatistics” for cohort studies [22], the infliximab study, which did not have a prospective subgroup design, included robust statistical models to address immortal time-bias, potential issues due to confounders and selection bias which were not considered by the other publications (Table 3).

### Noninterventional studies

Table 4 shows five noninterventional study scores using the CDA RWE checklist. Due to analogous study designs, the NA (not applicable) scores were similar for the five studies except for Dimethyl Fumarate and Tofacitinib, which showed a higher NA as both did not have study protocols. All studies used persistence as the study outcome, except dimethyl fumarate, which also included adherence as an additional endpoint. The Ustekinumab study in IBD patients [16] exhibited the highest compliance rate of 33 (lowest score in the not reported (NR) category), followed by three studies with the same score, erenumab for migraine treatment [20], tofacitinib for RA [18] and dimethyl Fumarate for MS [19], with the same NR score of 39. Ixekizumab in Plaque psoriasis [17] had the highest score of NR = 48.

**TABLE 4:** Noninterventional studies selected for evaluations.

CDA guidelines sections (items)	Ustekinumab in IBD^a^		Ixekizumab in PsA		Dimethyl fumarate in MS^a^		Tofacitinib in RA^a^		Erenumab in migraine	
Reporting scores	NR	NA	NR	NA	NR	NA	NR	NA	NR	NA
1. Study design and research questions [11]	0	1	2	1	3	1	3	1	3	1
2. Setting and content [4]	1	1	1	1	1	1	1	1	0	1
3. Data specifications, access, cleaning methods and linkage [10]	1	8	2	8	2	8	2	8	0	8
4. Data Sources, data dictionary and variables [12]	1	2	6	2	4	3	4	3	2	2
5. Participants [3]	3	2	6	2	2	2	3	2	4	2
6. Exposure definitions and comparators [8]	0	5	0	5	0	5	2	5	0	5
7. Outcomes [7]	2	1	1	1	0	1	1	1	1	1
8. Bias, confounding, and effect modifiers or subgroup effects [11]	9	0	11	0	11	0	11	0	10	0
9. Statistical Methods [5]	7	0	10	0	8	0	6	0	9	0
10. Study findings [8]	5	0	6	0	5	0	3	0	6	0
11. Interpretation and generalizability [7]	2	0	2	0	2	0	2	0	3	0
12. Limitations [2]	0	0	1	0	1	0	1	0	1	0
Total	31	19	48	19	39	20	39	20	39	19

NR, Not Reported; NA, Not Applicable.

^a^ With Supplementary Data published.

The Ustekinumab study [16] showed better compliance, illustrated by the lower scores in Sections 4 and 5, followed by 8, 9, and 11. Compared to the other three studies. Compliance with Section 4 was marked by the transparency of data sources, the availability of the database (Yale Open Data Access), data assessment on key variables before study initiation, and time-varying and continuous variables, which were reported with means and standard deviations. Compliance with section 5 was illustrated by a detailed description of the recruitment process and patients under study over time, a distinct protocol design diagram, details of exposure groups, discontinuation rules, and a definition of the study gap. All five studies had high NR scores in section 8 as confounders impacting assessment were not analyzed, and variables selected for regression analysis were controlled but not for confounders or missing data in section 9. Potential bias and confounders not evaluated in the study, limit the interpretation of the study findings.

Three studies, dimethyl fumarate, tofacitinib, and erenumab showed identical NR scores of 39. Erenumab scored higher in NR than Ustekinumab, mainly due to no study protocol *a priori*, lack of study governance, and no disclosure of a funding source. It was the only study that discussed the impact of bias on study results. Tofacitinib in RA lacked compliance, mainly in section 4, marked by inadequate data sources. The tofacitinib study also listed statistical packages and controlled variables under the statistical methods, which were not reported by the other two. None of the three studies mentioned data validity in terms of completeness or reliability, protocol design, or patient disposition. Confounders and biases were not mentioned in the study results or discussion. Ixekizumab had the lowest compliance mainly due to a lack of information on data sources, participants’ exposure details, and the issues mentioned for the other three products.

## Discussion

In the past few decades, the rise of specialty drugs, often requiring complex drug administrations such as infusions/injections or patient-safety monitoring, necessitated specialized patient assistance [10, 23]. In Canada, Patient Support programs (PSP) grew quickly in terms of numbers and level of service offered. In addition to drug administration and safety follow-up, staff from PSPs also help patients access reimbursement, provide education regarding the disease and the drug, and collect patient-level data to facilitate better patient management. An early report suggests that there are over 400 PSPs in Canada, each with the infrastructure to administer specialty drugs, provide patient care, and collect patient-level data [21]. A more in-depth analysis [24] at the drug level indicates that up to Aug 2023, of the 2556 prescription drugs marketed by 89 companies in Canada, 256 (10.0%) had a patient support program. Some drugs had multiple PSPs, and they were mostly managed by outside vendors.

The large and growing number of PSPs in Canada should serve as a gold mine for the generation of pan-Canadian RWE. Regulatory and HTA agencies consider data generated as part of a PSP to be a low level of evidence [8]. Most of these programs are tied to special drugs or companies, and the lack of public transparency in their operations is of high concern [10, 11]. Data quality remains obscure, including how missing data were handled, data completeness, data governance, and patient privacy. Databases used in PSPs are designed to collect information for patient management and not prospectively address scientific questions. How can that be used retrospectively to generate new effectiveness and safety information? Grundy et al., after analyzing a comprehensive database of PSPs in Canada up to August 2023, saw a strong correlation between drug prices and availability of PSPs, suggesting a commercial motive in the setting up of these PSPs besides patient care [24]. Due to these limitations, most PSP data publications focus on patients’ persistence and adherence to the drug tied to the specific PSP. For RWE from PSP data to be acceptable to regulatory agencies, additional rigor in transparency in design, data validation, and analysis are critical parameters.

With RWE gaining momentum in decision-making globally, manufacturers are interested in capturing and utilizing data from PSPs to gain additional insight into their products post-launch, such as effectiveness studies for reimbursement or supporting new indications. They are also aware that the databases currently structured are inadequate, and the data collection methodology would have to be revamped to meet the quality and transparency requirements matching those of clinical trials.

In collaboration with academic partners, regulatory agencies recently published checklists and templates to guide the generation of fit-for-purpose RWE intended for decision-making. The HARPER template [25], the STROBE checklist [26], and the CDA-AMC RWE checklist [8] were among the most recent ones. The HARPER template provides a set of core recommendations for clear and RWE protocols, whereas the STROBE checklist includes a list of high-level items that should be included in reports of cohort studies. The RWE checklist guide published by CDA-AMC is more prescriptive, specifying in detail the specific information required for each section. The CDA-AMC checklist has 12 sections, each with 2–12 items plus some with subitems. This publication evaluated nine peer-reviewed publications based on Canadian PSP databases using the CDA-AMC RWE checklist of 103 items (see Methods). The nine publications of cohort studies were divided into two groups, interventional and non-interventional, according to FDA RWE guidance [27]. The scores of NR (not reported) and NA (not applicable) were compared across studies. The NA scores serve as an indicator of the type of study design, highlighting that similarly designed studies will exhibit the same NA. On the other hand, NR scores measure the lack of compliance to critical parameters, which are deemed essential for a robust RWE study according to the CDA.

The infliximab study in IBD patients demonstrated the highest compliance with the lowest NR score among the interventional studies. The study was based on a protocol prospectively designed before the analysis (after data collection), with quality data checked and validated by a third party, a formal governance committee, ethics board approval, and justifications for missing data management. The study population and variables were well-defined and controlled, and study windows and gaps were specified. The Cox Proportional Hazards Model with the intervention time as the covariant was used to avoid immortal time bias. To account for measured and unmeasured confounders, quantitative bias analysis was used to evaluate critical endpoints. The study also completed a STROBE checklist and is in good standing. The study might still be inadequate in meeting all Health Canada and CDA-AMC requirements due to the lack of data transparency, as data extraction methods, code, algorithm, and data dictionary were not provided in the publication. The company probably considers the data information proprietary and would not disclose it in a publication, but would submit confidentially to the regulatory agencies. The rest of the interventional studies (vedolizumab in IBD, mepolizumab in asthma, and brodalumab in psoriasis) failed stringent data quality requirements, lacked statistical vigor, did not account for bias or confounders, and scored inadequately against the checklist. As for the non-interventional studies Ustekinumab in IBD, ixekiuzmab in psoriasis, dimethyl fumarate in MS, tofacitinib in RA, and erenumab in migraine, drug persistence was the objective of the studies, and endpoints were descriptive. The lack of control for bias and confounders might have skewed the data to be more favorable for the study drug; there was insufficient information for concluding, and these publications did not score favorably against the CDA-AMC checklist.

Limitations of the study: The most significant limitation was that the evaluations were performed on publications rather than study reports. These publications often omits details deemed confidential to manufacturers, restricting an assessment based on the checklist. The current paper attempts to perform the most precise evaluations based on publicly available information. The author believes that peer-reviewed publications serve as a good proxy for the quality of the study. Despite the need to protect confidential information, data quality validation, considerations of bias and confounders, and measures to control all key variables should be transparent in the publications. Without these details, the information cannot be used for decision-making. It is also acknowledged that most of the publications were written before the issuance of the CDA RWE guidance; however, the guidance was developed to align with established global standards, key principles for generating RWE were available for reference at the time these studies were conducted and should not have major impact on the quality of the studies.

## Conclusion

Nine published studies using PSP databases were analyzed using the CDA-AMC checklist. Only one study scored adequately to be a potential submission candidate for decision-making. For successful regulatory considerations, data quality validation, data source transparency, validated methodology to manage study bias, measured or unmeasured confounders, and robust outcome analysis, including sensitivity and quantitative bias analysis are critical factors to consider.

## Data Availability

Publicly available datasets were analyzed in this study.
