# Supplementary material for: A review to determine regulatorily and reimbursement successes of studies conducted using data from Canadian patient support programs based on the real-world evidence guidelines published by Canadian drug agency and health Canada
Source: J Pharm Pharm Sci. 2025 Aug 8;28:14587. doi: 10.3389/jpps.2025.14587 (PMC12371237; doi:10.3389/jpps.2025.14587)
Supplement: Supplementary file 1 [file DataSheet1.pdf]

Supplemental Table Content:	Pages
Table S1: Supplemental Table 1	2
(Disease Associations and the list of specialty medications with PSPs in Canada)	
Table S2: Infliximab in IBD	3
Table S3: Vedolizumab in IBD	11
Table S4: Brodalumab in Psoriasis	19
Table S5: Mepolizmab in Asthma	27
Table S6: Ustekinumab in IBD	35
Table S7: Ixekizumab in Psoriasis	43
Table S8: Tofacitinib in RA	51
Table S9: Dimethyl fumarate in MS	59
Table S10: Erenumab in migraine	67

Supplemental Table S1 (Disease Associations and the list of specialty medications with PSPs in Canada)

Disease Associations	Patient Support Programs source information	Disease types	Medications*	companies
Canadian Society of Intestinal Research<br><br>https://badgut.org/	IBD support programs when starting Biologics<br>https://badgut.org/information-centre/a-z-digestive-topics/patient-support-programs/	Inflammatory Bowel diseases	Infliximab<br>Adalimumab<br>Vedolizumab<br>Ustekinumab<br>Risankizumab	Janssen<br>AbbVie<br>Takeda<br>Janssen<br>AbbVie
Canadian Association of Rheumatoid Arthritis<br><br>https://arthritis.ca/treatment/your-patient-journey/rheumatoid-arthritis-en	https://rheuminfo.com/en/physician-tools/patient-support-program-enrolment-forms/	Rheumatoid arthritis	Certolizumab<br>Etanercept<br>Adalimumab<br>Apremilast<br>tofacitinib<br>Infliximab<br>Golimumab<br>Anakinra<br>Upadacitinib	UBC<br>Amgen<br>AbbVie<br>Celgene<br>Pfizer<br>Janssen<br>Janssen<br>Sobi<br>AbbVie
Canadian Associations of Psoriasis Patients	https://canadianpsoriasis.ca/en/treatment/medication-access/patient-support-programs	Psoriatic arthritis (PsA)<br>Psoriasis	Certolizumab<br>Secukinumab<br>Etanercept<br>Adalimumab<br>Apremilast<br>Infliximab<br>Golimumab<br>Ustekinumab<br>Brodalumab<br>Ixekizumab<br>Upadacitinib	UBC<br>Novartis<br>Amgen<br>AbbVie<br>Celgene<br>Janssen<br>Janssen<br>Janssen<br>Bausch<br>Lilly<br>AbbVie
Multiple Sclerosis MS Canada	https://mscanada.ca/health-information-lines-and-drug-support-programs	Multiple Sclerosis	Teriflunomide<br>Alemtuzumab<br>Interferon Beta -1a<br>Peg interferon -1a<br>Dimethyl Fumarate<br>Natalizumab<br>Glatiramer<br>Interferon Beta 1b<br>Fingolimod<br>Siponimod<br>Ofalumumab	Genzyme<br><br>Biogen<br><br>Biogen<br><br>Biogen<br><br>Biogen<br>Teva<br>Novartis<br><br>Novartis<br>Novartis<br>Novartis
Migraine Canada	https://migrainecanada.org/posts/the-migraine-tree/branches/preventive-treatments/cgrp-monoclonal-antibodies-cost-access-coverage/		erenumab<br>fremanezumab<br>galcanezumab<br>eptinezumab	Novartis<br>Teva<br>Lilly<br>Lundbeck
Canadian Severe Asthma Network	https://canadiansevereasthma.net/medications/biologics/		Mepolizumab (Nucala)<br>Reslizumab (Cinquair)<br>Benralizumab (Fasenra)	GSK<br><br>Teva<br><br>AstraZeneca

Supplemental Table S2 Infliximab in IBD (12) https://doi.org/10.1155/cjgh/5713315

Section	Check item	Reported on page number	If not reported or applicable, justify why
Section 1: Study design and research questions	1. Report a clearly stated am and study question	Pg 1<br>Sect :1	
	2. Report the overall study design	Pg 1, Sect 2	
	3. Provide a rationale for the choice of study design	Pg 1,2<br>Sect 1,2.1	
	4. Provide a relevant review of the literature to evaluate pertinent information and knowledge	Pg 2<br>Sect 1	
	5. Describe key elements of the study design (e.g., matching)	Pg 2<br>Sect 2.1	
	6. Consider the use of study diagrams to illustrate key aspects of the study design	Supple: pg 15	
	7. Strongly recommend to develop and reference an a priori protocol	Pg 2<br>Sect 2	
	8. Describe all study team members, including the role of patient partners, and any conflicts of interest	Pg 10-11	
	9. Describe the study governance structure, especially who was responsible for final decision-making	Pg 11	
	10. Report any research ethics approval (or equivalent)	Pg 2<br>Sect 1	
	11. Disclose sources of funding	Pg 11	
Section 2: Setting and Content	1. Describe important information to contextualize the data source, including:	Pg 2<br>Sect 2.1	
	1.1 type of care setting	Pg 2, Sect 2.1	
	1.2 geographic location		
	2. Describe all relevant study period dates, including periods of recruitment, exposure, follow-up, and data collection	Pg 2,3<br>Sect 2.1,2.2	
	3. Clearly identify missing data components in the data collection	Pg 2,5, Sect 2.1<br>Suppl pg 9-13	
	4. For studies that propose the use of a data source from a country other than Canada, Provide		NA

Section	Checklist item	Reported on page number(s)	If not reported or applicable, justify
	4.1 a rationale for selecting the data source		NA
	4.2 an explanation of how these factors might affect the generalizability of the study results to the population in Canada		NA
	4.3 background information about the healthcare system		NA
	4.4 description of prescribing and utilization practices		NA
Section 3: Data specifications-access, cleaning methods, and linkage	1. Describe the extent to which the investigation had access to database population used to create the study population and major aspects of data provenance	Pg 2 Section 2.1	
	2. Provide information on the data-cleaning methods used in the study. Share any data-cleaning code leveraged. If not, provided, justify	Pg 2 Section 2.1	
	3. Report whether data were organized by a Common Data Model structure		NA
	4. Describe the usage of data and consent for data sharing. Provide consent documents, if relevant		NA
	5. Describe data collection methods		NA
	6. Quality of the data and relevant metrics to assess the data quality should be reported		NA
	7. Describe any variability between data sources and the impact of changes over time in the data		NA
	8. Describe if any data linkage was conducted and the methods used for the linkage		NA
	9. Report who (e.g., which organization) performed the data linkage, if applicable		NA
	10. Describe the performance characteristics of the data linkage and the number of individuals linked at each stage of linkage		NA

	Checklist item	Reported on page number(s)	If not reported or applicable, justify
Secti0n 4: Data sources, data dictionary and variables	1. Provide and describe all data sources, including the specific version and date of the last update of the database	Pg 2<br>Section 2.1	
	2. Describe the characteristics of the health setting and context of data collection	Pg 2,3<br>Sect 2.1,2.2	
	3. Describe details of data continuity and completeness	Pg 5,6,7	
	4. Include the names, dates, and/or version numbers of when data were extracted for research use; by the data vendor or organization		NA
	5. Include the search and/or extraction criteria applied if the source data are a subset of the data from the vendor or organization, and provide calendar data ranges		NA
	6. Provide source(s)of data for each variable of interest		NR
	7. Describe how variables of interest were measured and if they have been adjudicated or validated in the population of interest	Pg 5-7<br>Sect<br>Suppl tables	
	8. Provide a data dictionary that includes information on data sources, validity and definitions for all variables, as applicable		NR
	9. Specify definitions and lookback windows for all variables	Pg 3<br>Sect 2.4	
	10. Report whether any variables could be time-varying (e.g., how the variable could change over time and when it was redefined in relation to time-varying exposures)	Pg 5-7 Sect 3.1-3.2<br>Supple tables	
	11. Report important variables that could not be captured and their anticipated impact on study results	Suppl 9-13<br>Supple tables	
	12. Provide information on deviations from a priori protocol in variable measurements	Supple tables	
Section 5: Participants	1. Provide inclusion criteria used to identify study population	Pg 2,3<br>Sect 2.2,2.3	
	2. Justify exclusion criteria and how they may affect the overall interpretation of the research	Pg 2,3<br>Sect 2.2	
	3. Describe study population characteristics relative to the target population in Canada	Pg 1,2<br>Secti 1	

Section	Checklist item	Reported on page number (s)	If not reported or applicable, justify why
	4. Provide all codes and algorithms used to define inclusions and exclusion criteria where possible		NR
	5. Specify the time period (e.g., lookback window) over which inclusion and exclusion criteria were assessed	Pg 2,3 Sect 2.2	
	6. Recommendations for specific study designs		
	6.1 For cohort studies, provide details leading to the analyzed cohort, including definitions for exposure groups, cohort entry and end dates matching criteria, and censoring/follow-up	Pg 3 Sect 2.4	
	6.2 For prospective cohort studies, describe recruitment processes	Pg 2 Sect 2.1	
	6.3 For case-controlled and case-crossover studies, proved details of case and control ascertainment, the source population for nested studies, sampling methods, and matching criteria		
	7. Report the number of participants at each stage of the study and reasons for nonparticipation. Consider illustrating this information using a flow diagram	Pg 14 Supporting info	
	8. Provide characteristics of study participants. If not available for feasible explain why	Pg 2,3 Sect 2.2,2.3	
	9. Indicate missing data for each variable of interest	Pg 2,5, Sect 2.1 Support info pg 9-13	
	10. Compare treatment or exposure groups		NA
	11. Specify the number of participants included in each analysis and the analysis strategy (e.g., per-protocol, ITT) and provide details on the number of proportion of subjects excluded from each analysis, and the reasons for exclusion	Pg 14 Supporting info	
Section 6: Exposure definitions and comparators	1. Define the requirements for the exposure definition (e.g./single, multiple, or continuous exposure) and relevant start and stop windows for assessing exposures	Pg 3 Sect 2.4	

Section	Checklist items	Reported on page number(s)	If not reported or not applicable. Justify why not
	2. Specify data source(s) from which exposure information was obtained, including validity and any limitations in exposure measurement	Pg 3<br>Sect 2.4	
	3. Specify the exposure-outcome risk window and discuss how it aligns with the known or anticipated relationship between the exposure and outcome timing	Pg 3<br>Sect 2.3,2.4	
	4. If no comparator was used, justify why not		NA
	5. Define the comparator group(s) (e.g., active comparator, historical comparator)		NA
	6. Provide justification for the comparator used, including potential implications and study design		NA
	7. Discuss any changes in patterns of use of the exposure and comparator(s) over time and how they may affect the results. Report any methods used to adjust for these changes.		NA
	8. Specify how adaptations to the intervention and/or comparator were permitted and recorded		NA
Section 7: Outcomes	1. Report definitions for all study outcomes (primary, secondary, and exploratory), where possible	Pg 4<br>Sect 3.1,3.2	
	2. Provide a rationale for the outcomes studied and discuss relevant outcomes not included in the study. Consider the use of a core outcome set if one is available for the condition of interest under study	Pg 10,11<br>Sect 4	
	3. Provide information about the validity of all outcome definitions	Pg 10,11<br>Sect 4	
	4. Describe whether the timing of the outcome can be accurately measured	Pg 10<br>Sect 4	
	5. Specify whether the outcome studied is a surrogate measure of a clinical (patient-centered) outcome and, if so, the strength of the relationship between the surrogate outcome and major clinical outcome(s)of interest	Pg 10<br>Sect 4	
	6. Discuss whether outcome misclassification could occur between treatment groups		NR

Section	Checklist item	Reported on page number(s)	If not reported or applicable, justify why not
	7. Report whether a control outcome was used and justify the control outcome(s)selected		NA
Section 8: Bias, confounding, and effect modifiers or subgroup effects	1. Report all procedures used to address potential sources of bias	Pg 2,3,4<br>2.4,2.5,3.1	
	2. Specify how potential sources of bias could influence the outcomes of the analysis	Suppl tables 19-22 a,b,c	
	3. Specify variables that were considered known or potential confounders in the analysis	Pg 4, 7-8, 10<br>Sect 3.1, 3.2, 4<br>supple:<br>Write-up pg 9-13,<br><br>Quantitative Bias analysis models: tables 19-22 a,b,c	
	4. Describe how confounder variables were selected and if they were informed of a causal diagram		
	5. Describe and compare the distribution of measured baseline confounding variables between treatment groups		
	6. Report whether any potential confounders could not be measured and specify the anticipated impact of these confounders on study results		
	7. Report whether time-varying confounding was considered and if not considered, why not		
	8. Specify the methods used to conduct assumptions and limitations of the data and, if no sensitivity analyses were conducted, explain why not		NA
	9. Specify known or potential effect modifiers		NR
	10. Describe any effect modification or subgroup analyses that were conducted and if they were specified a prior. Include if they were identified and conducted based on prespecified rationale. If no effect studies or biological rationale. If no effect modification or subgroup analyses were used, justify why they were not needed	Pg 3, 4, 7, 8,12<br>Sect 2.3, 3.2, 5<br>Fig 3-4	
	11. If effect modification or subgroup analyses were used, describe the methods and present separate results for each group		NR
Section 9: Statistical methods	1. Indicate the software used for the statistical analysis, including software package Version, and analytic tools employed (e.g., macros)	Pg 4<br>Sect 2.5	

Section	Checklist items	Report on page number(s)	If not reported or not applicable, justify why
	2. Provide access to the statistical code used or, if the code cannot be shared, explain why		NR
	3. Report all statistical methods used and justify their selection, including as applicable	Pg 3 Sect 2.5	
	3.1 all variables included in regression models	Pg 5,10, 3 Sect 2.4, 3.2, 5 Table 1	
	3.2 the method of variable selection for regression models		
	3.3 methods used to control for confounding	Refer to Sect 8.4 ABOVE	
	3.4 methods used for accounting for missing data	Pg 2, Sect 2.1, tables	
	3.5 how follow-up time and changes in exposures were handled	Pg 4, Sect 3.1,2, tables	
	3.6 subgroup analyses and effect modification	Ref to Sect 8.2 ABOVE	
	3.7 as applicable, stratification, propensity score estimation and assumptions, meta-analysis methods, validity of instrumental variables		NA
	4. Quantify the precision of all estimates using confidence intervals	Pg 3 Sect 2.5 Fig 2	
	5. Report the threshold of the statistical significance used	Pg 3, Sect 2.5, all tables, figs	
Section 10: Study findings	1. Summarize key results (estimated effect measures, measures of precision) with reference to each study objective and/or hypothesis for primary and secondary outcomes, and delineate these results by each treatment or exposure group	Pg 4-8 Sect 3.1-3.2 4 All tables and figures	
	2. Provide numbers of outcome events or summary measures of outcomes (or exposures in case-control studies)	Pg 4 Sect 3.1-3.2 All Suppl figs	
	3. Report both absolute and relative effect measures for binary outcomes, including their measure of precision		NR
	4. Report category boundaries when continuous variables are categorized and consider translating estimates of relative risk into absolute risk	Ref to Sect 8.4 and 9.5 ABOVE	
	5. Report unadjusted and adjusted estimates, including their measure of precision and confounders used for adjustment	Ref to Sect 8.3, 9.3.1 ABOVE	

Section	Checklist items	Reported on page number (s)	If not reported or not applicable, justify why
	6. Report other prespecified analyses conducted (e.g.,subgroup analyses interactions, sensitivity analyses)	Ref to Sect 8.3, 8.10 ABOVE	
	7. Describe any unplanned analyses performed secondarily (e.g., not defined a priori) and indicate these as exploratory		NR
	8. Avoid selecting reporting of results	Pg 3, sect 2.5	
Section 11: Interpretation and Generalizability	1. Provide an interpretation of the primary and secondary study results, as applicable	Pg 8-10 Sect 4	
	2. Interpret the findings from adjusted and unadjusted results as applicable	Pg 8-10 Sect 4	
	3. Discuss the Precision of the effect measure(s)		NR
	4. Discuss how potential biases and sensitivity of study assumptions may impact the results and subsequent interpretation	Pg 1,2,3 Sect 3.2, 4, 5	
	5. Discuss the implication of findings of clinical practice, including the risk-benefit profile of the treatment, if applicable	Pg 8-11 Sect 4, 5	
	6. Interpret study findings in relation to current literature	Pg 1,8-11 Sect 1, 4	
	7. Discuss the generalizability (external validity) of study results to the population in Canada	Pg 8-10 Sect 4	
Section 12: Limitations	1. Provide consideration of limitations of the study, including the data source, missing data, bias and confounding, imprecision or sample size limitations, and whether results are clinically meaningful	Pg 8-10 Sect 4	
	2. Discuss the plausibility of results and whether results could be due solely to chance, or confounding	Pg 10 Sect 4	

Supplemental Table: S3 vedolizumabin IBD (13) https://doi.org/10.1093/jcag/gwae010

Section	Checklist item	Reported on page number(s)	If not reported or not applicable, justify why
Section 1: Study design and research questions	12. Report a clearly stated aim and study question	290	
	13. Report the overall study design	291	
	14. Provide a rationale for the choice of study design	291	
	15. Provide a relevant review of the literature to evaluate pertinent information and knowledge	290, 297	
	16. Describe key elements of the study design (e.g., matching)	291	
	17. Consider the use of study diagrams to illustrate key aspects of the study design		NR
	18. Strongly recommend to develop and reference an a priori protocol	291	
	19. Describe all study team members, including the role of patient partners, and any conflicts of interest	290, 296	
	20. Describe the study governance structure, especially who was responsible for final decision-making	296	
	21. Report any research ethics approval (or equivalent)	291	
	22. Disclose sources of funding	296	
Section 2: Setting and Content	5. Describe important information to contextualize the data source, including:	296	
	1.1 type of care setting	291	
	1.2 geographic location	291	
	6. Describe all relevant study period dates, including periods of recruitment, exposure, follow-up, and data collection	291	
	7. Clearly identify missing data components in the data collection		NR
	8. For studies that propose the use of a data source from a country other than Canada, Provide		NA

Section	Checklist item	Reported on page number(s)	If not reported or not applicable, justify why
	4.1 a rationale for selecting the data source		NA
	4.2 an explanation of how these factors might affect the generalizability of the study results to the population in Canada		NA
	4.3 background information about the healthcare system		NA
	4.4 description of prescribing and utilization practices		NA
Section 3: Data specifications-access, cleaning methods, and linkage	11. Describe the extent to which the investigation had access to database population used to create the study population and major aspects of data provenance	297	
	12. Provide information on the data-cleaning methods used in the study. Share any data-cleaning code leveraged. If not, provided, justify		NR
	13. Report whether data were organized by a Common Data Model structure		NA
	14. Describe the usage of data and consent for data sharing. Provide consent documents, if relevant		NA
	15. Describe data collection methods		NA
	16. Quality of the data and relevant metrics to assess the data quality should be reported		NA
	17. Describe any variability between data sources and the impact of changes over time in the data		NA
	18. Describe if any data linkage was conducted and the methods used for the linkage		NA
	19. Report who (e.g., which organization) performed the data linkage, if applicable		NA
	20. Describe the performance characteristics of the data linkage and the number of individuals linked at each stage of linkage		NA

Section	Checklist item	Reported on page number(s)	If not reported or not applicable, justify why
Secti0n 4: Data sources, data dictionary and variables	13. Provide and describe all data sources, including the specific version and date of the last update of the database	297	
	14. Describe the characteristics of the health setting and context of data collection	291	
	15. Describe details of data continuity and completeness	291	
	16. Include the names, dates, and/or version numbers of when data were extracted for research use; by the data vendor or organization	292	
	17. Include the search and/or extraction criteria applied if the source data are a subset of the data from the vendor or organization, and provide calendar data ranges		NA
	18. Provide source(s)of data for each variable of interest		NA
	19. Describe how variables of interest were measured and if they have been adjudicated or validated in the population of interest	291, 295-296	
	20. Provide a data dictionary that includes information on data sources, validity and definitions for all variables, as applicable		NR
	21. Specify definitions and lookback windows for all variables	292	
	22. Report whether any variables could be time-varying (e.g., how the variable could change over time and when it was redefined in relation to time-varying exposures)	291-295	
	23. Report important variables that could not be captured and their anticipated impact on study results	291-295	
	24. Provide information on deviations from a priori protocol in variable measurements		NR
Section 5: Participants	12. Provide inclusion criteria used to identify study population	291	
	13. Justify exclusion criteria and how they may affect the overall interpretation of the research	291	
	14. Describe study population characteristics relative to the target population in Canada	291	

Section	Checklist item	Reported on page number (s)	If not reported or not applicable, justify why
	15. Provide all codes and algorithms used to define inclusions and exclusion criteria where possible		NR
	16. Specify the time period (e.g., lookback window) over which inclusion and exclusion criteria were assessed	291	
	17. Recommendations for specific study designs	291-292	
	6.1 For cohort studies, provide details leading to the analyzed cohort, including definitions for exposure groups, cohort entry and end dates matching criteria, and censoring/follow-up	291	
	6.2 For prospective cohort studies, describe recruitment processes		
	6.3 For case-controlled and case-crossover studies, proved details of case and control ascertainment, the source population for nested studies, sampling methods, and matching criteria		
	18. Report the number of participants at each stage of the study and reasons for nonparticipation. Consider illustrating this information using a flow diagram	292-293,295	
	19. Provide characteristics of study participants. If not available for feasible explain why	292, 296	
	20. Indicate missing data for each variable of interest		NR
	21. Compare treatment or exposure groups		NA
	22. Specify the number of participants included in each analysis and the analysis strategy (e.g., per-protocol, ITT) and provide details on the number of proportion of subjects excluded from each analysis, and the reasons for exclusion		NR
Section 6: Exposure definitions and comparators	2. Define the requirements for the exposure definition (e.g./single, multiple, or continuous exposure) and relevant start and stop windows for assessing exposures	291-292	

Section	Checklist items	Reported on page number(s)	If not reported or not applicable. Justify why
	9. Specify data source(s) from which exposure information was obtained, including validity and any limitations in exposure measurement	291-291	
	10. Specify the exposure-outcome risk window and discuss how it aligns with the known or anticipated relationship between the exposure and outcome timing	291-291	
	11. If no comparator was used, justify why not		NA
	12. Define the comparator group(s) (e.g., active comparator, historical comparator)		NA
	13. Provide justification for the comparator used, including potential implications and study design		NA
	14. Discuss any changes in patterns of us of the exposure and comparator(s) over time and how they may affect the results. Report any methods used to adjust for these changes.		NA
	15. Specify how adaptations to the intervention and/or comparator were permitted and recorded		NA
Section 7: Outcomes	8. Report definitions for all study outcomes (primary, secondary, and exploratory), where possible	292	
	9. Provide a rationale for the outcomes studied and discuss relevant outcomes not included in the study. Consider the use of a core outcome set if one is available for the condition of interest under study	292	
	10. Provide information about the validity of all outcome definitions		NR
	11. Describe whether the timing of the outcome can be accurately measured	292	
	12. Specify whether the outcome studied is a surrogate measure of a clinical (patient-centered) outcome and, if so, the strength of the relationship between the surrogate outcome and major clinical outcome(s)of interest	292	
	13. Discuss whether outcome misclassification could occur between treatment groups		NR

Section	Checklist item	Reported on page number(s)	If not reported or not applicable, justify why
	14. Report whether a control outcome was used and justify the control outcome(s)selected		NA
Section 8: Bias, confounding, and effect modifiers or subgroup effects	12. Report all procedures used to address potential sources of bias		NR
	13. Specify how potential sources of bias could influence the outcomes of the analysis		NR
	14. Specify variables that were considered known or potential confounders in the analysis		NR
	15. Describe how confounder variables were selected and if they were informed of a causal diagram		NR
	16. Describe and compare the distribution of measured baseline confounding variables between treatment groups		NR
	17. Report whether any potential confounders could not be measured and specify the anticipated impact of these confounders on study results		NR
	18. Report whether time-varying confounding was considered and if not considered, why not		NR
	19. Specify the methods used to conduct assumptions and limitations of the data and, if no sensitivity analyses were conducted, explain why not		NA
	20. Specify known or potential effect modifiers		NR
	21. Describe any effect modification or subgroup analyses that were conducted and if they were specified a prior. Include if they were identified and conducted based on prespecified rationale. If no effect studies or biological rationale. If no effect modification or subgroup analyses were used, justify why they were not needed		NR
	22. If effect modification or subgroup analyses were used, describe the methods and present separate results for each group		NR
Section 9: Statistical methods	9. Indicate the software used for the statistical analysis, including software package version, and analytic tools employed (e.g., macros)	291	

Section	Checklist items	Report on page number(s)	If not reported or not applicable, justify why
	10. Provide access to the statistical code used or, if the code cannot be shared, explain why		NR
	11. Report all statistical methods used and justify their selection, including as applicable		
	3.1 all variables included in regression models	291-295	
	3.2 the method of variable selection for regression models	291	
	3.3 methods used to control for confounding		NR
	3.4 methods used for accounting for missing data	291	
	3.5 how follow-up time and changes in exposures were handled		NR
	3.6 subgroup analyses and effect modification	292-295	
	3.7 as applicable, stratification, propensity score estimation and assumptions, meta-analysis methods, validity of instrumental variables		NA
	12. Quantify the precision of all estimates using confidence intervals	291-295	
	13. Report the threshold of the statistical significance used	291-295<br>S1-S3	
Section 10: Study findings	6. Summarize key results (estimated effect measures, measures of precision) with reference to each study objective and/or hypothesis for primary and secondary outcomes, and delineate these results by each treatment or exposure group	295-296	
	7. Provide numbers of outcome events or summary measures of outcomes (or exposures in case-control studies)	294-295<br>S1-S3	
	8. Report both absolute and relative effect measures for binary outcomes, including their measure of precision	294-295	
	9. Report category boundaries when continuous variables are categorized and consider translating estimates of relative risk into absolute risk	291-295<br>S1-S3	
	10. Report unadjusted and adjusted estimates, including their measure of precision and confounders used for adjustment		NR

Section	Checklist items	Reported on page number (s)	If not reported or not applicable, justify why
	14. Report other prespecified analyses conducted (e.g., subgroup analyses interactions, sensitivity analyses)	296	
	15. Describe any unplanned analyses performed secondarily (e.g., not defined a priori) and indicate these as exploratory		NR
	16. Avoid selecting reporting of results		NR
Section 11: Interpretation and Generalizability	8. Provide an interpretation of the primary and secondary study results, as applicable	295-296	
	9. Interpret the findings from adjusted and unadjusted results as applicable		NR
	10. Discuss the Precision of the effect measure(s)		NR
	11. Discuss how potential biases and sensitivity of study assumptions may impact the results and subsequent interpretation	291	
	12. Discuss the implication of findings of clinical practice, including the risk-benefit profile of the treatment, if applicable	295-296	
	13. Interpret study findings in relation to current literature	295-296	
	14. Discuss the generalizability (external validity) of study results to the population in Canada	295-296	
Section 12: Limitations	3. Provide consideration of limitations of the study, including the data source, missing data, bias and confounding, imprecision or sample size limitations, and whether results are clinically meaningful	296	
	4. Discuss the plausibility of results and whether results could be due solely to chance, or confounding	296	

Supplemental Table S4 : Brodalumab in Psoriasis (14)
https://doi.org/10.1177/12034754231168851

Section	Check item	Reported on page number	If not reported or applicable, justify why
Section 1 : Study design and research questions	23. Report a clearly stated aim and study question	226-227	
	24. Report the overall study design	227	
	25. Provide a rationale for the choice of study design	226-227	
	26. Provide a relevant review of the literature to evaluate pertinent information and knowledge	226, 231	
	27. Describe key elements of the study design (e.g., matching)	227	
	28. Consider the use of study diagrams to illustrate key aspects of the study design		NR
	29. Strongly recommend to develop and reference an a priori protocol		NR
	30. Describe all study team members, including the role of patient partners, and any conflicts of interest	223	
	31. Describe the study governance structure, especially who was responsible for final decision-making		NR
	32. Report any research ethics approval (or equivalent)		NR
	33. Disclose sources of funding	233	
Section 2: Setting and Content	9. Describe important information to contextualize the data source, including:		
	1.1 type of care setting	227	
	1.2 geographic location	226	
	10. Describe all relevant study period dates, including periods of recruitment, exposure, follow-up, and data collection	227	
	11. Clearly identify missing data components in the data collection		NR
	12. For studies that propose the use of a data source from a country other than Canada, Provide		NA

Section	Checklist item	Reported on page number(s)	If not reported or applicable, justify why
	4.1 a rationale for selecting the data source		NA
	4.2 an explanation of how these factors might affect the generalizability of the study results to the population in Canada		NA
	4.3 background information about the healthcare system		NA
	4.4 description of prescribing and utilization practices		NA
Section 3: Data specifications-access, cleaning methods, and linkage	21. Describe the extent to which the investigation had access to database population used to create the study population and major aspects of data provenance		NR
	22. Provide information on the data-cleaning methods used in the study. Share any data-cleaning code leveraged. If not, provided, justify		NR
	23. Report whether data were organized by a Common Data Model structure		NA
	24. Describe the usage of data and consent for data sharing. Provide consent documents, if relevant		NA
	25. Describe data collection methods		NA
	26. Quality of the data and relevant metrics to assess the data quality should be reported		NA
	27. Describe any variability between data sources and the impact of changes over time in the data		NA
	28. Describe if any data linkage was conducted and the methods used for the linkage		NA
	29. Report who (e.g., which organization) performed the data linkage, if applicable		NA
	30. Describe the performance characteristics of the data linkage and the numberof individuals linked at each stage of linkage		NA

	Checklist item	Reported on page number(s)	If not reported or applicable, justify why
Section 4: Data sources, data dictionary and variables	25. Provide and describe all data sources, including the specific version and date of the last update of the database		NR
	26. Describe the characteristics of the health setting and context of data collection	291	
	27. Describe details of data continuity and completeness	291	
	28. Include the names, dates, and/or version numbers of when data were extracted for research use; by the data vendor or organization		NA
	29. Include the search and/or extraction criteria applied if the source data are a subset of the data from the vendor or organization, and provide calendar data ranges		NA
	30. Provide source(s)of data for each variable of interest		NR
	31. Describe how variables of interest were measured and if they have been adjudicated or validated in the population of interest		NR
	32. Provide a data dictionary that includes information on data sources, validity and definitions for all variables, as applicable		NR
	33. Specify definitions and lookback windows for all variables	227	
	34. Report whether any variables could be time-varying (e.g., how the variable could change over time and when it was redefined in relation to time-varying exposures)	227	
	35. Report important variables that could not be captured and their anticipated impact on study results	291-295	
	36. Provide information on deviations from a priori protocol in variable measurements		NR
Section 5: Participants	23. Provide inclusion criteria used to identify study population	227	
	24. Justify exclusion criteria and how they may affect the overall interpretation of the research		NR
	25. Describe study population characteristics relative to the target population in Canada	227	

Section	Checklist item	Reported on page number (s)	If not reported or applicable, justify why
	26. Provide all codes and algorithms used to define inclusions and exclusion criteria where possible		NR
	27. Specify the time period (e.g., lookback window) over which inclusion and exclusion criteria were assessed	227	
	28. Recommendations for specific study designs		
	6.1 For cohort studies, provide details leading to the analyzed cohort, including definitions for exposure groups, cohort entry and end dates matching criteria, and censoring/follow-up	227-230	
	6.2 For prospective cohort studies, describe recruitment processes		
	6.3 For case-controlled and case-crossover studies, proved details of case and control ascertainment, the source population for nested studies, sampling methods, and matching criteria		
	29. Report the number of participants at each stage of the study and reasons for nonparticipation. Consider illustrating this information using a flow diagram	227-230	
	30. Provide characteristics of study participants. If not available for feasible explain why	227	
	31. Indicate missing data for each variable of interest		NR
	32. Compare treatment or exposure groups		NA
	33. Specify the number of participants included in each analysis and the analysis strategy (e.g., per-protocol, ITT) and provide details on the number of proportion of subjects excluded from each analysis, and the reasons for exclusion	227-230	
Section 6: Exposure definitions and comparators	3. Define the requirements for the exposure definition (e.g./single, multiple, or continuous exposure) and relevant start and stop windows for assessing exposures	227	

Section	Checklist items	Reported on page number(s)	If not reported or not applicable. Justify why
	16. Specify data source(s) from which exposure information was obtained, including validity and any limitations in exposure measurement	227	
	17. Specify the exposure-outcome risk window and discuss how it aligns with the known or anticipated relationship between the exposure and outcome timing	227-230	
	18. If no comparator was used, justify why not		NA
	19. Define the comparator group(s) (e.g., active comparator, historical comparator)		NA
	20. Provide justification for the comparator used, including potential implications and study design		NA
	21. Discuss any changes in patterns of use of the exposure and comparator(s) over time and how they may affect the results. Report any methods used to adjust for these changes.		NA
	22. Specify how adaptations to the intervention and/or comparator were permitted and recorded		NA
Section 7: Outcomes	15. Report definitions for all study outcomes (primary, secondary, and exploratory), where possible	227-233	
	16. Provide a rationale for the outcomes studied and discuss relevant outcomes not included in the study. Consider the use of a core outcome set if one is available for the condition of interest under study	227-233	
	17. Provide information about the validity of all outcome definitions	227-228	
	18. Describe whether the timing of the outcome can be accurately measured	230	
	19. Specify whether the outcome studied is a surrogate measure of a clinical (patient-centered) outcome and, if so, the strength of the relationship between the surrogate outcome and major clinical outcome(s)of interest	226-227	
	20. Discuss whether outcome misclassification could occur between treatment groups		NR

Section	Checklist item	Reported on page number(s)	If not reported or applicable, justify why
	21. Report whether a control outcome was used and justify the control outcome(s)selected		NA
Section 8: Bias, confounding, and effect modifiers or subgroup effects	23. Report all procedures used to address potential sources of bias		NR
	24. Specify how potential sources of bias could influence the outcomes of the analysis		NR
	25. Specify variables that were considered known or potential confounders in the analysis		NR
	26. Describe how confounder variables were selected and if they were informed of a causal diagram		NR
	27. Describe and compare the distribution of measured baseline confounding variables between treatment groups		NR
	28. Report whether any potential confounders could not be measured and specify the anticipated impact of these confounders on study results		NR
	29. Report whether time-varying confounding was considered and if not considered, why not		NR
	30. Specify the methods used to conduct assumptions and limitations of the data and, if no sensitivity analyses were conducted, explain why not		NR
	31. Specify known or potential effect modifiers		NR
	32. Describe any effect modification or subgroup analyses that were conducted and if they were specified a prior. Include if they were identified and conducted based on prespecified rationale. If no effect studies or biological rationale. If no effect modification or subgroup analyses were used, justify why they were not needed		NR
	33. If effect modification or subgroup analyses were used, describe the methods and present separate results for each group		NR
Section 9: Statistical methods	17. Indicate the software used for the statistical analysis, including software package version, and analytic tools employed (e.g., macros)	227	

Section	Checklist items	Report on page number(s)	If not reported or not applicable, justify why
	18. Provide access to the statistical code used or, if the code cannot be shared, explain why		NR
	19. Report all statistical methods used and justify their selection, including as applicable		
	3.1 all variables included in regression models	291,294-295	
	3.2 the method of variable selection for regression models	291	
	3.3 methods used to control for confounding		NR
	3.4 methods used for accounting for missing data	291	
	3.5 how follow-up time and changes in exposures were handled	229-233	
	3.6 subgroup analyses and effect modification	291-295<br>S1-S3	
	3.7 as applicable, stratification, propensity score estimation and assumptions, meta-analysis methods, validity of instrumental variables		NA
	20. Quantify the precision of all estimates using confidence intervals		NR
	21. Report the threshold of the statistical significance used	291	
Section 10: Study findings	11. Summarize key results (estimated effect measures, measures of precision) with reference to each study objective and/or hypothesis for primary and secondary outcomes, and delineate these results by each treatment or exposure group	227,233	
	12. Provide numbers of outcome events or summary measures of outcomes (or exposures in case-control studies)	227,233	
	13. Report both absolute and relative effect measures for binary outcomes, including their measure of precision		NR
	14. Report category boundaries when continuous variables are categorized and consider translating estimates of relative risk into absolute risk		NR
	15. Report unadjusted and adjusted estimates, including their measure of precision and confounders used for adjustment		NR

Section	Checklist items	Reported on page number (s)	If not reported or not applicable, justify why
	22. Report other prespecified analyses conducted (e.g.,subgroup analyses interactions, sensitivity analyses)		NR
	23. Describe any unplanned analyses performed secondarily (e.g., not defined a priori) and indicate these as exploratory		NR
	24. Avoid selecting reporting of results		NR
Section 11: Interpretation and Generalizability	15. Provide an interpretation of the primary and secondary study results, as applicable	230-233	
	16. Interpret the findings from adjusted and unadjusted results as applicable		NR
	17. Discuss the Precision of the effect measure(s)		NR
	18. Discuss how potential biases and sensitivity of study assumptions may impact the results and subsequent interpretation	232	
	19. Discuss the implication of findings of clinical practice, including the risk-benefit profile of the treatment, if applicable	232-233	
	20. Interpret study findings in relation to current literature	230-233	
	21. Discuss the generalizability (external validity) of study results to the population in Canada	232	
Section 12: Limitations	5. Provide consideration of limitations of the study, including the data source, missing data, bias and confounding, imprecision or sample size limitations, and whether results are clinically meaningful	232	
	6. Discuss the plausibility of results and whether results could be due solely to chance, or confounding	232	

Supplemental Table S5: Mepolizumab in Asthma (15) https://doi.org/10.1186/s13223-023-00863-7

Section	Checklist item	Reported on page number(s)	If not reported or not applicable, justify why
Section 1: Study design and research questions	34. Report a clearly stated am and study question	2	
	35. Report the overall study design	2-3	
	36. Provide a rationale for the choice of study design	3	
	37. Provide a relevant review of the literature to evaluate pertinent information and knowledge	2,7-8	
	38. Describe key elements of the study design (e.g., matching)	3	
	39. Consider the use of study diagrams to illustrate key aspects of the study design	5	
	40. Strongly recommend to develop and reference an a priori protocol	4	
	41. Describe all study team members, including the role of patient partners, and any conflicts of interest	1,10	
	42. Describe the study governance structure, especially who was responsible for final decision-making	10	
	43. Report any research ethics approval (or equivalent)	10	
	44. Disclose sources of funding	10	
Section 2: Setting and Content	13. Describe important information to contextualize the data source, including:	2-3	
	1.1 type of care setting	2-3	
	1.2 geographic location	2	
	14. Describe all relevant study period dates, including periods of recruitment, exposure, follow-up, and data collection	2	
	15. Clearly identify missing data components in the data collection	4	
	16. For studies that propose the use of a data source from a country other than Canada, Provide		NA

Section	Checklist item	Reported on page number(s)	If not reported or not applicable, justify why
	4.1 a rationale for selecting the data source		NA
	4.2 an explanation of how these factors might affect the generalizability of the study results to the population in Canada		NA
	4.3 background information about the healthcare system		NA
	4.4 description of prescribing and utilization practices		NA
Section 3: Data specifications-access, cleaning methods, and linkage	31. Describe the extent to which the investigation had access to database population used to create the study population and major aspects of data provenance	2-4	
	32. Provide information on the data-cleaning methods used in the study. Share any data-cleaning code leveraged. If not, provided, justify		NR
	33. Report whether data were organized by a Common Data Model structure		NA
	34. Describe the usage of data and consent for data sharing. Provide consent documents, if relevant	2-3	
	35. Describe data collection methods	2-3	
	36. Quality of the data and relevant metrics to assess the data quality should be reported		
	37. Describe any variability between data sources and the impact of changes over time in the data	2,5	
	38. Describe if any data linkage was conducted and the methods used for the linkage	2-3,5	
	39. Report who (e.g., which organization) performed the data linkage, if applicable	10	
	40. Describe the performance characteristics of the data linkage and the number of individuals linked at each stage of linkage	2-3,5	

Section	Checklist item	Reported on page number(s)	If not reported or not applicable, justify why
Secti0n 4: Data sources, data dictionary and variables	37. Provide and describe all data sources, including the specific version and date of the last update of the database	2-3	
	38. Describe the characteristics of the health setting and context of data collection	2,8-9	
	39. Describe details of data continuity and completeness	2-3	
	40. Include the names, dates, and/or version numbers of when data were extracted for research use; by the data vendor or organization	10	
	41. Include the search and/or extraction criteria applied if the source data are a subset of the data from the vendor or organization, and provide calendar data ranges	2,10	
	42. Provide source(s)of data for each variable of interest	2,10	
	43. Describe how variables of interest were measured and if they have been adjudicated or validated in the population of interest	3-4	
	44. Provide a data dictionary that includes information on data sources, validity and definitions for all variables, as applicable	2-3, 9	
	45. Specify definitions and lookback windows for all variables	2	
	46. Report whether any variables could be time-varying (e.g., how the variable could change over time and when it was redefined in relation to time-varying exposures)	3-4	
	47. Report important variables that could not be captured and their anticipated impact on study results	3-4	
	48. Provide information on deviations from a priori protocol in variable measurements		NR
Section 5: Participants	34. Provide inclusion criteria used to identify study population	2-3	
	35. Justify exclusion criteria and how they may affect the overall interpretation of the research	2,4	
	36. Describe study population characteristics relative to the target population in Canada	7,9	

Section	Checklist item	Reported on page number (s)	If not reported or not applicable, justify why
	37. Provide all codes and algorithms used to define inclusions and exclusion criteria where possible		NA
	38. Specify the time period (e.g., lookback window) over which inclusion and exclusion criteria were assessed	2	
	39. Recommendations for specific study designs		
	6.1 For cohort studies, provide details leading to the analyzed cohort, including definitions for exposure groups, cohort entry and end dates matching criteria, and censoring/follow-up	2, 5	
	6.2 For prospective cohort studies, describe recruitment processes		
	6.3 For case-controlled and case-crossover studies, proved details of case and control ascertainment, the source population for nested studies, sampling methods, and matching criteria		
	40. Report the number of participants at each stage of the study and reasons for nonparticipation. Consider illustrating this information using a flow diagram	5	
	41. Provide characteristics of study participants. If not available for feasible explain why	2-5	
	42. Indicate missing data for each variable of interest	4	
	43. Compare treatment or exposure groups	6-8	
	44. Specify the number of participants included in each analysis and the analysis strategy (e.g., per-protocol, ITT) and provide details on the number of proportion of subjects excluded from each analysis, and the reasons for exclusion		NR
Section 6: Exposure definitions and comparators	4. Define the requirements for the exposure definition (e.g./single, multiple, or continuous exposure) and relevant start and stop windows for assessing exposures	2-5	

Section	Checklist items	Reported on page number(s)	If not reported or not applicable. Justify why
	23. Specify data source(s) from which exposure information was obtained, including validity and any limitations in exposure measurement	2, 10	
	24. Specify the exposure-outcome risk window and discuss how it aligns with the known or anticipated relationship between the exposure and outcome timing	7-9	
	25. If no comparator was used, justify why not		NA
	26. Define the comparator group(s) (e.g., active comparator, historical comparator)		NA
	27. Provide justification for the comparator used, including potential implications and study design		NA
	28. Discuss any changes in patterns of us of the exposure and comparator(s) over time and how they may affect the results. Report any methods used to adjust for these changes.		NA
	29. Specify how adaptations to the intervention and/or comparator were permitted and recorded		NA
Section 7: Outcomes	22. Report definitions for all study outcomes (primary, secondary, and exploratory), where possible	6-7	
	23. Provide a rationale for the outcomes studied and discuss relevant outcomes not included in the study. Consider the use of a core outcome set if one is available for the condition of interest under study	6-9	
	24. Provide information about the validity of all outcome definitions	6-7	
	25. Describe whether the timing of the outcome can be accurately measured	6-8	
	26. Specify whether the outcome studied is a surrogate measure of a clinical (patient-centered) outcome and, if so, the strength of the relationship between the surrogate outcome and major clinical outcome(s)of interest	7-9	
	27. Discuss whether outcome misclassification could occur between treatment groups	9	

Section	Checklist item	Reported on page number(s)	If not reported or not applicable, justify why
	28. Report whether a control outcome was used and justify the control outcome(s)selected		NA
Section 8: Bias, confounding, and effect modifiers or subgroup effects	34. Report all procedures used to address potential sources of bias		NR
	35. Specify how potential sources of bias could influence the outcomes of the analysis		NR
	36. Specify variables that were considered known or potential confounders in the analysis		NR
	37. Describe how confounder variables were selected and if they were informed of a causal diagram		NR
	38. Describe and compare the distribution of measured baseline confounding variables between treatment groups		NR
	39. Report whether any potential confounders could not be measured and specify the anticipated impact of these confounders on study results		NR
	40. Report whether time-varying confounding was considered and if not considered, why not		NR
	41. Specify the methods used to conduct assumptions and limitations of the data and, if no sensitivity analyses were conducted, explain why not		NR
	42. Specify known or potential effect modifiers		NR
	43. Describe any effect modification or subgroup analyses that were conducted and if they were specified a prior. Include if they were identified and conducted based on prespecified rationale. If no effect studies or biological rationale. If no effect modification or subgroup analyses were used, justify why they were not needed		NR
	44. If effect modification or subgroup analyses were used, describe the methods and present separate results for each group		NR
Section 9: Statistical methods	25. Indicate the software used for the statistical analysis, including software package version, and analytic tools employed (e.g., macros)	3-4	

Section	Checklist items	Report on page number(s)	If not reported or not applicable, justify why
	26. Provide access to the statistical code used or, if the code cannot be shared, explain why	3-4, 10	
	27. Report all statistical methods used and justify their selection, including as applicable		
	3.1 all variables included in regression models	3-4	
	3.2 the method of variable selection for regression models	3-4	
	3.3 methods used to control for confounding		NR
	3.4 methods used for accounting for missing data		NR
	3.5 how follow-up time and changes in exposures were handled		NR
	3.6 subgroup analyses and effect modification	3-4	
	3.7 as applicable, stratification, propensity score estimation and assumptions, meta-analysis methods, validity of instrumental variables		NA
	28. Quantify the precision of all estimates using confidence intervals	6-8	
	29. Report the threshold of the statistical significance used	6-8<br>S2, S3	
Section 10: Study findings	16. Summarize key results (estimated effect measures, measures of precision) with reference to each study objective and/or hypothesis for primary and secondary outcomes, and delineate these results by each treatment or exposure group	4-7	
	17. Provide numbers of outcome events or summary measures of outcomes (or exposures in case-control studies)	4-9<br>S1-S3	
	18. Report both absolute and relative effect measures for binary outcomes, including their measure of precision	4-7	
	19. Report category boundaries when continuous variables are categorized and consider translating estimates of relative risk into absolute risk	4-9<br>S2,S3	
	20. Report unadjusted and adjusted estimates, including their measure of precision and confounders used for adjustment		NR

Section	Checklist items	Reported on page number (s)	If not reported or not applicable, justify why
	30. Report other prespecified analyses conducted (e.g., subgroup analyses interactions, sensitivity analyses)	2-3	
	31. Describe any unplanned analyses performed secondarily (e.g., not defined a priori) and indicate these as exploratory		NR
	32. Avoid selecting reporting of results		NR
Section 11: Interpretation and Generalizability	22. Provide an interpretation of the primary and secondary study results, as applicable	4-8	
	23. Interpret the findings from adjusted and unadjusted results as applicable		NR
	24. Discuss the Precision of the effect measure(s)	8	
	25. Discuss how potential biases and sensitivity of study assumptions may impact the results and subsequent interpretation		NR
	26. Discuss the implication of findings of clinical practice, including the risk-benefit profile of the treatment, if applicable	7-9	
	27. Interpret study findings in relation to current literature	7-8	
	28. Discuss the generalizability (external validity) of study results to the population in Canada	7-9	
Section 12: Limitations	7. Provide consideration of limitations of the study, including the data source, missing data, bias and confounding, imprecision or sample size limitations, and whether results are clinically meaningful	3,9	
	8. Discuss the plausibility of results and whether results could be due solely to chance, or confounding	9	

Supplemental Table S6: Ustekinumab in IBD (16)https://doi.org/10.1007/s12325-023-02611-0

Section	Check item	Reported on page number	If not reported or applicable, justify why
Section 1: Study design and research questions	45. Report a clearly stated am and study question	4423	
	46. Report the overall study design	4423	
	47. Provide a rationale for the choice of study design	4423	
	48. Provide a relevant review of the literature to evaluate pertinent information and knowledge	4421-4423	
	49. Describe key elements of the study design (e.g., matching)		NA
	50. Consider the use of study diagrams to illustrate key aspects of the study design	Suppl Fig 1	
	51. Strongly recommend to develop and reference an a priori protocol	4423	
	52. Describe all study team members, including the role of patient partners, and any conflicts of interest	4437	
	53. Describe the study governance structure, especially who was responsible for final decision-making	4437	
	54. Report any research ethics approval (or equivalent)	4423, 4438	
	55. Disclose sources of funding	4437	
Section 2: Setting and Content	17. Describe important information to contextualize the data source, including:		
	1.1 type of care setting	4427	
	1.2 geographic location	4423	
	18. Describe all relevant study period dates, including periods of recruitment, exposure, follow-up, and data collection	4427	
	19. Clearly identify missing data components in the data collection		NR
	20. For studies that propose the use of a data source from a country other than Canada, Provide		NA

Section	Checklist item	Reported on page number(s)	If not reported or applicable, justify
	4.1 a rationale for selecting the data source		NA
	4.2 an explanation of how these factors might affect the generalizability of the study results to the population in Canada		NA
	4.3 background information about the healthcare system		NA
	4.4 description of prescribing and utilization practices		NA
Section 3: Data specifications- access, cleaning methods, and linkage	41. Describe the extent to which the investigation had access to database population used to create the study population and major aspects of data provenance	4437	
	42. Provide information on the data-cleaning methods used in the study. Share any data-cleaning code leveraged. If not, provided, justify		NR
	43. Report whether data were organized by a Common Data Model structure		NA
	44. Describe the usage of data and consent for data sharing. Provide consent documents, if relevant		NA
	45. Describe data collection methods		NA
	46. Quality of the data and relevant metrics to assess the data quality should be reported		NA
	47. Describe any variability between data sources and the impact of changes over time in the data		NA
	48. Describe if any data linkage was conducted and the methods used for the linkage		NA
	49. Report who (e.g., which organization) performed the data linkage, if applicable		NA
	50. Describe the performance characteristics of the data linkage and the number of individuals linked at each stage of linkage		NA

	Checklist item	Reported on page number(s)	If not reported or applicable, justify
Secti0n 4: Data sources, data dictionary and variables	49. Provide and describe all data sources, including the specific version and date of the last update of the database	4437	
	50. Describe the characteristics of the health setting and context of data collection	4423-4424	
	51. Describe details of data continuity and completeness	Suppl Tab 1	
	52. Include the names, dates, and/or version numbers of when data were extracted for research use; by the data vendor or organization		NA
	53. Include the search and/or extraction criteria applied if the source data are a subset of the data from the vendor or organization, and provide calendar data ranges		NA
	54. Provide source(s)of data for each variable of interest	4424	
	55. Describe how variables of interest were measured and if they have been adjudicated or validated in the population of interest	4425	
	56. Provide a data dictionary that includes information on data sources, validity and definitions for all variables, as applicable		NR
	57. Specify definitions and lookback windows for all variables	4424, Suppl Fig 1	
	58. Report whether any variables could be time-varying (e.g., how the variable could change over time and when it was redefined in relation to time-varying exposures)	4425	
	59. Report important variables that could not be captured and their anticipated impact on study results	4425	
	60. Provide information on deviations from a priori protocol in variable measurements		NA
Section 5: Participants	45. Provide inclusion criteria used to identify study population	4427	
	46. Justify exclusion criteria and how they may affect the overall interpretation of the research		NR
	47. Describe study population characteristics relative to the target population in Canada	Suppl Tab 1	

Section	Checklist item	Reported on page number (s)	If not reported or applicable, justify why
	48. Provide all codes and algorithms used to define inclusions and exclusion criteria where possible		NR
	49. Specify the time period (e.g., lookback window) over which inclusion and exclusion criteria were assessed	4427	
	50. Recommendations for specific study designs	Suppl Fig 1	
	6.1 For cohort studies, provide details leading to the analyzed cohort, including definitions for exposure groups, cohort entry and end dates matching criteria, and censoring/follow-up	4425	
	6.2 For prospective cohort studies, describe recruitment processes	4427	
	6.3 For case-controlled and case-crossover studies, proved details of case and control ascertainment, the source population for nested studies, sampling methods, and matching criteria		
	51. Report the number of participants at each stage of the study and reasons for nonparticipation. Consider illustrating this information using a flow diagram	Supp Tab 1	
	52. Provide characteristics of study participants. If not available for feasible explain why	4424	
	53. Indicate missing data for each variable of interest		NR
	54. Compare treatment or exposure groups		NA
	55. Specify the number of participants included in each analysis and the analysis strategy (e.g., per-protocol, ITT) and provide details on the number of proportion of subjects excluded from each analysis, and the reasons for exclusion	Suppl Tab 1	
Section 6: Exposure definitions and comparators	5. Define the requirements for the exposure definition (e.g./single, multiple, or continuous exposure) and relevant start and stop windows for assessing exposures	4424	

Section	Checklist items	Reported on page number(s)	If not reported or not applicable. Justify why not
	30. Specify data source(s) from which exposure information was obtained, including validity and any limitations in exposure measurement	4437	
	31. Specify the exposure-outcome risk window and discuss how it aligns with the known or anticipated relationship between the exposure and outcome timing	4424-4425	
	32. If no comparator was used, justify why not		NA
	33. Define the comparator group(s) (e.g., active comparator, historical comparator)		NA
	34. Provide justification for the comparator used, including potential implications and study design		NA
	35. Discuss any changes in patterns of us of the exposure and comparator(s) over time and how they may affect the results. Report any methods used to adjust for these changes.		NA
	36. Specify how adaptations to the intervention and/or comparator were permitted and recorded		NA
Section 7: Outcomes	29. Report definitions for all study outcomes (primary, secondary, and exploratory), where possible	4432-4433	
	30. Provide a rationale for the outcomes studied and discuss relevant outcomes not included in the study. Consider the use of a core outcome set if one is available for the condition of interest under study	4432-4433	
	31. Provide information about the validity of all outcome definitions	4425	
	32. Describe whether the timing of the outcome can be accurately measured		NR
	33. Specify whether the outcome studied is a surrogate measure of a clinical (patient-centered) outcome and, if so, the strength of the relationship between the surrogate outcome and major clinical outcome(s)of interest	4432-4433	
	34. Discuss whether outcome misclassification could occur between treatment groups		NR

Section	Checklist item	Reported on page number(s)	If not reported or applicable, justify why not
	35. Report whether a control outcome was used and justify the control outcome(s)selected		NA
Section 8: Bias, confounding, and effect modifiers or subgroup effects	45. Report all procedures used to address potential sources of bias		NR
	46. Specify how potential sources of bias could influence the outcomes of the analysis		NR
	47. Specify variables that were considered known or potential confounders in the analysis	4437	
	48. Describe how confounder variables were selected and if they were informed of a causal diagram		NR
	49. Describe and compare the distribution of measured baseline confounding variables between treatment groups		NR
	50. Report whether any potential confounders could not be measured and specify the anticipated impact of these confounders on study results		NR
	51. Report whether time-varying confounding was considered and if not considered, why not	4437	
	52. Specify the methods used to conduct assumptions and limitations of the data and, if no sensitivity analyses were conducted, explain why not		NR
	53. Specify known or potential effect modifiers		NR
	54. Describe any effect modification or subgroup analyses that were conducted and if they were specified aprior. Include if they were identified and conducted based on prespecified rationale. If no effect studies or biological rationale. If no effect modification or subgroup analyses were used, justify why they were not needed		NR
	55. If effect modification or subgroup analyses were used, describe the methods and present separate results for each group		NR
Section 9: Statistical methods	33. Indicate the software used for the statistical analysis, including software package Version, and analytic tools employed (e.g., macros)		NR

Section	Checklist items	Report on page number(s)	If not reported or not applicable, justify why
	34. Provide access to the statistical code used or, if the code cannot be shared, explain why	Suppl Figs 3-5	
	35. Report all statistical methods used and justify their selection, including as applicable	4425-4428	
	3.1 all variables included in regression models	4425	
	3.2 the method of variable selection for regression models	4425	
	3.3 methods used to control for confounding		NR
	3.4 methods used for accounting for missing data		NR
	3.5 how follow-up time and changes in exposures were handled		NR
	3.6 subgroup analyses and effect modification	4425	
	3.7 as applicable, stratification, propensity score estimation and assumptions, meta-analysis methods, validity of instrumental variables		NR
	36. Quantify the precision of all estimates using confidence intervals		NR
	37. Report the threshold of the statistical significance used		NR
Section 10: Study findings	21. Summarize key results (estimated effect measures, measures of precision) with reference to each study objective and/or hypothesis for primary and secondary outcomes, and delineate these results by each treatment or exposure group	4428-4436	
	22. Provide numbers of outcome events or summary measures of outcomes (or exposures in case-control studies)	4427-4435	
	23. Report both absolute and relative effect measures for binary outcomes, including their measure of precision		NR
	24. Report category boundaries when continuous variables are categorized and consider translating estimates of relative risk into absolute risk		NR
	25. Report unadjusted and adjusted estimates, including their measure of precision and confounders used for adjustment	Suppl Fig 3-5	

Section	Checklist items	Reported on page number (s)	If not reported` ` ` ` ` ` ` ` or not applicable, justify why
	38. Report other prespecified analyses conducted (e.g.,subgroup analyses interactions, sensitivity analyses)		NR
	39. Describe any unplanned analyses performed secondarily (e.g., not defined a priori) and indicate these as exploratory		NR
	40. Avoid selecting reporting of results		NR
Section 11: Interpretation and Generalizability	29. Provide an interpretation of the primary and secondary study results, as applicable	4432-4437	
	30. Interpret the findings from adjusted and unadjusted results as applicable		NR
	31. Discuss the Precision of the effect measure(s)		NR
	32. Discuss how potential biases and sensitivity of study assumptions may impact the results and subsequent interpretation	4437	
	33. Discuss the implication of findings of clinical practice, including the risk-benefit profile of the treatment, if applicable	4432-4436	
	34. Interpret study findings in relation to current literature	4432-4437	
	35. Discuss the generalizability (external validity) of study results to the population in Canada	4432-4437	
Section 12: Limitations	9. Provide consideration of limitations of the study, including the data source, missing data, bias and confounding, imprecision or sample size limitations, and whether results are clinically meaningful	4437	
	10. Discuss the plausibility of results and whether results could be due solely to chance, or confounding	4437	

Supplement Table S7: Ixekizumab in plaque psoriasis (17) https://doi.org/10.1007/s13555-022-00853-4

Section	Check item	Reported on page number	If not reported or applicable, justify why
Section 1 : Study design and research questions	56. Report a clearly stated am and study question	236	
	57. Report the overall study design	236	
	58. Provide a rationale for the choice of study design	236	
	59. Provide a relevant review of the literature to evaluate pertinent information and knowledge	236-237	
	60. Describe key elements of the study design (e.g., matching)		NA
	61. Consider the use of study diagrams to illustrate key aspects of the study design		NR
	62. Strongly recommend to develop and reference an a priori protocol		NR
	63. Describe all study team members, including the role of patient partners, and any conflicts of interest	241	
	64. Describe the study governance structure, especially who was responsible for final decision-making	241	
	65. Report any research ethics approval (or equivalent)	237	
	66. Disclose sources of funding	241	
Section 2: Setting and Content	21. Describe important information to contextualize the data source, including:		
	1.1 type of care setting	237	
	1.2 geographic location	237	
	22. Describe all relevant study period dates, including periods of recruitment, exposure, follow-up, and data collection	237	
	23. Clearly identify missing data components in the data collection		NR
	24. For studies that propose the use of a data source from a country other than Canada, Provide		NA

Section	Checklist item	Reported on page number(s)	If not reported or applicable, justify
	4.1 a rationale for selecting the data source		NA
	4.2 an explanation of how these factors might affect the generalizability of the study results to the population in Canada		NA
	4.3 background information about the healthcare system		NA
	4.4 description of prescribing and utilization practices		NA
Section 3: Data specifications- access, cleaning methods, and linkage	51. Describe the extent to which the investigation had access to database population used to create the study population and major aspects of data provenance		NR
	52. Provide information on the data-cleaning methods used in the study. Share any data-cleaning code leveraged. If not, provided, justify		NR
	53. Report whether data were organized by a Common Data Model structure		NA
	54. Describe the usage of data and consent for data sharing. Provide consent documents, if relevant		NA
	55. Describe data collection methods		NA
	56. Quality of the data and relevant metrics to assess the data quality should be reported		NA
	57. Describe any variability between data sources and the impact of changes over time in the data		NA
	58. Describe if any data linkage was conducted and the methods used for the linkage		NA
	59. Report who (e.g., which organization) performed the data linkage, if applicable		NA
	60. Describe the performance characteristics of the data linkage and the numberof individuals linked at each stage of linkage		NA

	Checklist item	Reported on page number(s)	If not reported or applicable, justify
Section 4: Data sources, data dictionary and variables	61. Provide and describe all data sources, including the specific version and date of the last update of the database	237,243	
	62. Describe the characteristics of the health setting and context of data collection	237	
	63. Describe details of data continuity and completeness	237-238	
	64. Include the names, dates, and/or version numbers of when data were extracted for research use; by the data vendor or organization		NA
	65. Include the search and/or extraction criteria applied if the source data are a subset of the data from the vendor or organization, and provide calendar data ranges		NA
	66. Provide source(s)of data for each variable of interest		NR
	67. Describe how variables of interest were measured and if they have been adjudicated or validated in the population of interest	237	
	68. Provide a data dictionary that includes information on data sources, validity and definitions for all variables, as applicable		NR
	69. Specify definitions and lookback windows for all variables		NR
	70. Report whether any variables could be time-varying (e.g., how the variable could change over time and when it was redefined in relation to time-varying exposures)	238	
	71. Report important variables that could not be captured and their anticipated impact on study results		NR
	72. Provide information on deviations from a priori protocol in variable measurements		NR
Section 5: Participants	56. Provide inclusion criteria used to identify study population	237	
	57. Justify exclusion criteria and how they may affect the overall interpretation of the research	237	
	58. Describe study population characteristics relative to the target population in Canada	236	

Section	Checklist item	Reported on page number (s)	If not reported or applicable, justify why
	59. Provide all codes and algorithms used to define inclusions and exclusion criteria where possible		NR
	60. Specify the time period (e.g., lookback window) over which inclusion and exclusion criteria were assessed		NR
	61. Recommendations for specific study designs		NR
	6.1 For cohort studies, provide details leading to the analyzed cohort, including definitions for exposure groups, cohort entry and end dates matching criteria, and censoring/follow-up	237	
	6.2 For prospective cohort studies, describe recruitment processes	236-237	
	6.3 For case-controlled and case-crossover studies, proved details of case and control ascertainment, the source population for nested studies, sampling methods, and matching criteria		NA
	62. Report the number of participants at each stage of the study and reasons for nonparticipation. Consider illustrating this information using a flow diagram		NR
	63. Provide characteristics of study participants. If not available for feasible explain why	237	
	64. Indicate missing data for each variable of interest		NR
	65. Compare treatment or exposure groups		NA
	66. Specify the number of participants included in each analysis and the analysis strategy (e.g., per-protocol, ITT) and provide details on the number of proportion of subjects excluded from each analysis, and the reasons for exclusion		NR
Section 6: Exposure definitions and comparators	6. Define the requirements for the exposure definition (e.g./single, multiple, or continuous exposure) and relevant start and stop windows for assessing exposures	236-237	

Section	Checklist items	Reported on page number(s)	If not reported or not applicable. Justify why not
	37. Specify data source(s) from which exposure information was obtained, including validity and any limitations in exposure measurement	243	
	38. Specify the exposure-outcome risk window and discuss how it aligns with the known or anticipated relationship between the exposure and outcome timing	238-239	
	39. If no comparator was used, justify why not		NA
	40. Define the comparator group(s) (e.g., active comparator, historical comparator)		NA
	41. Provide justification for the comparator used, including potential implications and study design		NA
	42. Discuss any changes in patterns of us of the exposure and comparator(s) over time and how they may affect the results. Report any methods used to adjust for these changes.		NA
	43. Specify how adaptations to the intervention and/or comparator were permitted and recorded		NA
Section 7: Outcomes	36. Report definitions for all study outcomes (primary, secondary, and exploratory), where possible	237	
	37. Provide a rationale for the outcomes studied and discuss relevant outcomes not included in the study. Consider the use of a core outcome set if one is available for the condition of interest under study	237	
	38. Provide information about the validity of all outcome definitions		NR
	39. Describe whether the timing of the outcome can be accurately measured	240	
	40. Specify whether the outcome studied is a surrogate measure of a clinical (patient-centered) outcome and, if so, the strength of the relationship between the surrogate outcome and major clinical outcome(s)of interest	238-239	
	41. Discuss whether outcome misclassification could occur between treatment groups	239-240	

Section	Checklist item	Reported on page number(s)	If not reported or applicable, justify why not
	42. Report whether a control outcome was used and justify the control outcome(s)selected		NA
Section 8: Bias, confounding, and effect modifiers or subgroup effects	56. Report all procedures used to address potential sources of bias		NR
	57. Specify how potential sources of bias could influence the outcomes of the analysis		NR
	58. Specify variables that were considered known or potential confounders in the analysis		NR
	59. Describe how confounder variables were selected and if they were informed of a causal diagram		NR
	60. Describe and compare the distribution of measured baseline confounding variables between treatment groups		NR
	61. Report whether any potential confounders could not be measured and specify the anticipated impact of these confounders on study results		NR
	62. Report whether time-varying confounding was considered and if not considered, why not		NR
	63. Specify the methods used to conduct assumptions and limitations of the data and, if no sensitivity analyses were conducted, explain why not		NR
	64. Specify known or potential effect modifiers		NR
	65. Describe any effect modification or subgroup analyses that were conducted and if they were specified a prior. Include if they were identified and conducted based on prespecified rationale. If no effect studies or biological rationale. If no effect modification or subgroup analyses were used, justify why they were not needed		NR
	66. If effect modification or subgroup analyses were used, describe the methods and present separate results for each group		NR
Section 9: Statistical methods	41. Indicate the software used for the statistical analysis, including software package Version, and analytic tools employed (e.g., macros)		NR

Section	Checklist items	Report on page number(s)	If not reported or not applicable, justify why
	42. Provide access to the statistical code used or, if the code cannot be shared, explain why		NR
	43. Report all statistical methods used and justify their selection, including as applicable		
	3.1 all variables included in regression models		NR
	3.2 the method of variable selection for regression models	238	
	3.3 methods used to control for confounding		NR
	3.4 methods used for accounting for missing data		NR
	3.5 how follow-up time and changes in exposures were handled		NR
	3.6 subgroup analyses and effect modification		NR
	3.7 as applicable, stratification, propensity score estimation and assumptions, meta-analysis methods, validity of instrumental variables		NR
	44. Quantify the precision of all estimates using confidence intervals		NR
	45. Report the threshold of the statistical significance used		NR
Section 10: Study findings	26. Summarize key results (estimated effect measures, measures of precision) with reference to each study objective and/or hypothesis for primary and secondary outcomes, and delineate these results by each treatment or exposure group	238-239	
	27. Provide numbers of outcome events or summary measures of outcomes (or exposures in case-control studies)	239-239	
	28. Report both absolute and relative effect measures for binary outcomes, including their measure of precision		NR
	29. Report category boundaries when continuous variables are categorized and consider translating estimates of relative risk into absolute risk		NR
	30. Report unadjusted and adjusted estimates, including their measure of precision and confounders used for adjustment		NR

Section	Checklist items	Reported on page number (s)	If not report or not applicable, justify why
	46. Report other prespecified analyses conducted (e.g.,subgroup analyses interactions, sensitivity analyses)		NR
	47. Describe any unplanned analyses performed secondarily (e.g., not defined a priori) and indicate these as exploratory		NR
	48. Avoid selecting reporting of results		NR
Section 11: Interpretation and Generalizability	36. Provide an interpretation of the primary and secondary study results, as applicable	238-241	
	37. Interpret the findings from adjusted and unadjusted results as applicable		NR
	38. Discuss the Precision of the effect measure(s)		NR
	39. Discuss how potential biases and sensitivity of study assumptions may impact the results and subsequent interpretation	241	
	40. Discuss the implication of findings of clinical practice, including the risk-benefit profile of the treatment, if applicable	240	
	41. Interpret study findings in relation to current literature	238-241	
	42. Discuss the generalizability (external validity) of study results to the population in Canada	243	
Section 12: Limitations	11. Provide consideration of limitations of the study, including the data source, missing data, bias and confounding, imprecision or sample size limitations, and whether results are clinically meaningful	243	
	12. Discuss the plausibility of results and whether results could be due solely to chance, or confounding		NR

Supplemental Table S8: Tofacitinib in RA (18) https://doi.org/10.1093/rheumatology/kez324

Section	Check item	Reported on page number(s)	If not reported or applicable, justify why
Section 1 : Study design and research questions	67. Report a clearly stated am and study question	569	
	68. Report the overall study design	569	
	69. Provide a rationale for the choice of study design	569	
	70. Provide a relevant review of the literature to evaluate pertinent information and knowledge	571	
	71. Describe key elements of the study design (e.g., matching)		NA
	72. Consider the use of study diagrams to illustrate key aspects of the study design		NR
	73. Strongly recommend to develop and reference an a priori protocol		NR
	74. Describe all study team members, including the role of patient partners, and any conflicts of interest	573	
	75. Describe the study governance structure, especially who was responsible for final decision-making		NR
	76. Report any research ethics approval (or equivalent)	569	
	77. Disclose sources of funding	573	
Section 2: Setting and Content	25. Describe important information to contextualize the data source, including:		
	1.1 type of care setting	568,569	
	1.2 geographic location	568	
	26. Describe all relevant study period dates, including periods of recruitment, exposure, follow-up, and data collection	569	
	27. Clearly identify missing data components in the data collection		NR
	28. For studies that propose the use of a data source from a country other than Canada, Provide		NA

Section	Checklist item	Reported on page number(s)	If not reported or applicable, justify
	4.1 a rationale for selecting the data source	569	
	4.2 an explanation of how these factors might affect the generalizability of the study results to the population in Canada		NA
	4.3 background information about the healthcare system		NA
	4.4 description of prescribing and utilization practices		NA
Section 3: Data specifications-access, cleaning methods, and linkage	61. Describe the extent to which the investigation had access to database population used to create the study population and major aspects of data provenance	569	
	62. Provide information on the data-cleaning methods used in the study. Share any data-cleaning code leveraged. If not, provided, justify		NR
	63. Report whether data were organized by a Common Data Model structure		NA
	64. Describe the usage of data and consent for data sharing. Provide consent documents, if relevant		NA
	65. Describe data collection methods		NA
	66. Quality of the data and relevant metrics to assess the data quality should be reported		NR
	67. Describe any variability between data sources and the impact of changes over time in the data		NA
	68. Describe if any data linkage was conducted and the methods used for the linkage		NA
	69. Report who (e.g., which organization) performed the data linkage, if applicable		NA
	70. Describe the performance characteristics of the data linkage and the number of individuals linked at each stage of linkage		NA

	Checklist item	Reported on page number(s)	If not reported or applicable, justify
Secti0n 4: Data sources, data dictionary and variables	73. Provide and describe all data sources, including the specific version and date of the last update of the database	569	
	74. Describe the characteristics of the health setting and context of data collection	569	
	75. Describe details of data continuity and completeness	571	
	76. Include the names, dates, and/or version numbers of when data were extracted for research use; by the data vendor or organization		NA
	77. Include the search and/or extraction criteria applied if the source data are a subset of the data from the vendor or organization, and provide calendar data ranges		NA
	78. Provide source(s)of data for each variable of interest		NR
	79. Describe how variables of interest were measured and if they have been adjudicated or validated in the population of interest	569, 572	
	80. Provide a data dictionary that includes information on data sources, validity and definitions for all variables, as applicable		NR
	81. Specify definitions and lookback windows for all variables	571-572	NR
	82. Report whether any variables could be time-varying (e.g., how the variable could change over time and when it was redefined in relation to time-varying exposures)	571-572	NR
	83. Report important variables that could not be captured and their anticipated impact on study results	571	
	84. Provide information on deviations from a priori protocol in variable measurements		NA
Section 5: Participants	67. Provide inclusion criteria used to identify the study population	569	
	68. Justify exclusion criteria and how they may affect the overall interpretation of the research	569	
	69. Describe study population characteristics relative to the target population in Canada	569,571	

Section	Checklist item	Reported on page number (s)	If not reported or applicable, justify why
	70. Provide all codes and algorithms used to define inclusions and exclusion criteria where possible		NR
	71. Specify the time period (e.g., lookback window) over which inclusion and exclusion criteria were assessed	569,571	
	72. Recommendations for specific study designs		NA
	6.1 For cohort studies, provide details leading to the analyzed cohort, including definitions for exposure groups, cohort entry and end dates matching criteria, and censoring/follow-up		NA
	6.2 For prospective cohort studies, describe recruitment processes		NA
	6.3 For case-controlled and case-crossover studies, proved details of case and control ascertainment, the source population for nested studies, sampling methods, and matching criteria		NA
	73. Report the number of participants at each stage of the study and reasons for nonparticipation. Consider illustrating this information using a flow diagram	569,571	
	74. Provide characteristics of study participants. If not available for feasible explain why	569	
	75. Indicate missing data for each variable of interest		NR
	76. Compare treatment or exposure groups		NA
	77. Specify the number of participants included in each analysis and the analysis strategy (e.g., per-protocol, ITT) and provide details on the number of proportion of subjects excluded from each analysis, and the reasons for exclusion		NR
Section 6: Exposure definitions and comparators	7. Define the requirements for the exposure definition (e.g./single, multiple, or continuous exposure) and relevant start and stop windows for assessing exposures	569	

Section	Checklist items	Reported on page number(s)	If not reported or not applicable. Justify why not
	44. Specify data source(s) from which exposure information was obtained, including validity and any limitations in exposure measurement		NR
	45. Specify the exposure-outcome risk window and discuss how it aligns with the known or anticipated relationship between the exposure and outcome timing		NR
	46. If no comparator was used, justify why not		NA
	47. Define the comparator group(s) (e.g., active comparator, historical comparator)		NA
	48. Provide justification for the comparator used, including potential implications and study design		NA
	49. Discuss any changes in patterns of us of the exposure and comparator(s) over time and how they may affect the results. Report any methods used to adjust for these changes.		NA
	50. Specify how adaptations to the intervention and/or comparator were permitted and recorded		NA
Section 7: Outcomes	43. Report definitions for all study outcomes (primary, secondary, and exploratory), where possible	560-570	
	44. Provide a rationale for the outcomes studied and discuss relevant outcomes not included in the study. Consider the use of a core outcome set if one is available for the condition of interest under study	571, 573	
	45. Provide information about the validity of all outcome definitions		NR
	46. Describe whether the timing of the outcome can be accurately measured	569,571	
	47. Specify whether the outcome studied is a surrogate measure of a clinical (patient-centered) outcome and, if so, the strength of the relationship between the surrogate outcome and major clinical outcome(s)of interest	571	
	48. Discuss whether outcome misclassification could occur between treatment groups	571	

Section	Checklist item	Reported on page number(s)	If not reported or applicable, justify why not
	49. Report whether a control outcome was used and justify the control outcome(s)selected		NA
Section 8: Bias, confounding, and effect modifiers or subgroup effects	67. Report all procedures used ot address potential sources of bias		NR
	68. Specify how potential sources of bias could influence the outcomes of the analysis		NR
	69. Specify variables that were considered known or potential confounders in the analysis		NR
	70. Describe how confounder variables were selected and if they were informed of a causal diagram		NR
	71. Describe and compare the distribution of measured baseline confounding variables between treatment groups		NR
	72. Report whether any potential confounders could not be measured and specify the anticipated impact of these confounders on study results		NR
	73. Report whether time-varying confounding was considered and if not considered, why not		NR
	74. Specify the methods used to conduct assumptions and limitations of the data and, if no sensitivity analyses were conducted, explain why not		NR
	75. Specify known or potential effect modifiers		NR
	76. Describe any effect modification or subgroup analyses that were conducted and if they were specified a prior. Include if they were identified and conducted based on prespecified rationale. If no effect studies or biological rationale. If no effect modification or subgroup analyses were used, justify why they were not needed		NR
	77. If effect modification or subgroup analyses were used, describe the methods and present separate results for each group		NR
Section 9: Statistical methods	49. Indicate the software used for the statistical analysis, including software package Version, and analytic tools employed (e.g., macros)	569	

Section	Checklist items	Report on page number(s)	If not reported or not applicable, justify why
	50. Provide access to the statistical code used or, if the code cannot be shared, explain why	569	
	51. Report all statistical methods used and justify their selection, including as applicable		
	3.1 all variables included in regression models		NR
	3.2 the method of variable selection for regression models	569	
	3.3 methods used to control for confounding		NR
	3.4 methods used for accounting for missing data		NR
	3.5 how follow-up time and changes in exposures were handled	569, 571	
	3.6 subgroup analyses and effect modification		NR
	3.7 as applicable, stratification, propensity score estimation and assumptions, meta-analysis methods, validity of instrumental variables		NR
	52. Quantify the precision of all estimates using confidence intervals		NR
	53. Report the threshold of the statistical significance used	569	
Section 10: Study findings	31. Summarize key results (estimated effect measures, measures of precision) with reference to each study objective and/or hypothesis for primary and secondary outcomes, and delineate these results by each treatment or exposure group	572	
	32. Provide numbers of outcome events or summary measures of outcomes (or exposures in case-control studies)	570-572 Suppl tables 1-3	
	33. Report both absolute and relative effect measures for binary outcomes, including their measure of precision	573, Suppl tables 1-3	
	34. Report category boundaries when continuous variables are categorized and consider translating estimates of relative risk into absolute risk	573	
	35. Report unadjusted and adjusted estimates, including their measure of precision and confounders used for adjustment	573	

Section	Checklist items	Reported on page number (s)	If not reported or not applicable, justify why
	54. Report other prespecified analyses conducted (e.g.,subgroup analyses interactions, sensitivity analyses)		NR
	55. Describe any unplanned analyses performed secondarily (e.g., not defined a priori) and indicate these as exploratory		NR
	56. Avoid selecting reporting of results		NR
Section 11: Interpretation and Generalizability	43. Provide an interpretation of the primary and secondary study results, as applicable	571-573	
	44. Interpret the findings from adjusted and unadjusted results as applicable		NR
	45. Discuss the Precision of the effect measure(s)	572	
	46. Discuss how potential biases and sensitivity of study assumptions may impact the results and subsequent interpretation		NR
	47. Discuss the implication of findings of clinical practice, including the risk-benefit profile of the treatment, if applicable	571,573	
	48. Interpret study findings in relation to current literature	571,573	
	49. Discuss the generalizability (external validity) of study results to the population in Canada	571,573	
Section 12: Limitations	13. Provide consideration of limitations of the study, including the data source, missing data, bias and confounding, imprecision or sample size limitations, and whether results are clinically meaningful	571	
	14. Discuss the plausibility of results and whether results could be due solely to chance, or confounding		NR

Supplemental Table S9: Dimethly fumarate in MS (19)
https://doi.org/10.1016/j.msard.2022.104080

Section	Check item	Reported on page number	If not reported or applicable, justify why
Section 1 : Study design and research questions	78. Report a clearly stated am and study question	Pg 1<br>Sect 2.2	
	79. Report the overall study design	Pg 2, Sect 2	
	80. Provide a rationale for the choice of study design	Pg 2<br>Sect 1	
	81. Provide a relevant review of the literature to evaluate pertinent information and knowledge	Pg 6-7<br>Sect 4	
	82. Describe key elements of the study design (e.g., matching)		NA
	83. Consider the use of study diagrams to illustrate key aspects of the study design		NR
	84. Strongly recommend to develop and reference an a priori protocol		NR
	85. Describe all study team members, including the role of patient partners, and any conflicts of interest	Pg 7-8<br>Sect 4	
	86. Describe the study governance structure, especially who was responsible for final decision-making		NR
	87. Report any research ethics approval (or equivalent)	Pg 8<br>Sect 4	
	88. Disclose sources of funding	Pg 8, sect 4	
Section 2: Setting and Content	29. Describe important information to contextualize the data source, including:		
	1.1 type of care setting	Pg 2, sect 2.2	
	1.2 geographic location	Pg 2 sect 1	
	30. Describe all relevant study period dates, including periods of recruitment, exposure, follow-up, and data collection	Pg 2<br>Sect 2.3	
	31. Clearly identify missing data components in the data collection		NR
	32. For studies that propose the use of a data source from a country other than Canada, Provide		NA

Section	Checklist item	Reported on page number(s)	If not reported or applicable, justify
	4.1 a rationale for selecting the data source		NA
	4.2 an explanation of how these factors might affect the generalizability of the study results to the population in Canada		NA
	4.3 background information about the healthcare system		NA
	4.4 description of prescribing and utilization practices		NA
Section 3: Data specifications-access, cleaning methods, and linkage	71. Describe the extent to which the investigation had access to database population used to create the study population and major aspects of data provenance	Pg 2 Section2.3	
	72. Provide information on the data-cleaning methods used in the study. Share any data-cleaning code leveraged. If not, provided, justify		NR
	73. Report whether data were organized by a Common Data Model structure		NA
	74. Describe the usage of data and consent for data sharing. Provide consent documents, if relevant		NA
	75. Describe data collection methods		NA
	76. Quality of the data and relevant metrics to assess the data quality should be reported		NR
	77. Describe any variability between data sources and the impact of changes over time in the data		NA
	78. Describe if any data linkage was conducted and the methods used for the linkage		NA
	79. Report who (e.g., which organization) performed the data linkage, if applicable		NA
	80. Describe the performance characteristics of the data linkage and the number of individuals linked at each stage of linkage		NA

	Checklist item	Reported on page number(s)	If not reported or applicable, justify
Section 4: Data sources, data dictionary and variables	85. Provide and describe all data sources, including the specific version and date of the last update of the database	Pg 2 Section 2.3	
	86. Describe the characteristics of the health setting and context of data collection	Pg 2 Sect 2.1,2.2	
	87. Describe details of data continuity and completeness	Pg 3 Sect 3.1	
	88. Include the names, dates, and/or version numbers of when data were extracted for research use; by the data vendor or organization		NA
	89. Include the search and/or extraction criteria applied if the source data are a subset of the data from the vendor or organization, and provide calendar data ranges		NA
	90. Provide source(s)of data for each variable of interest		NR
	91. Describe how variables of interest were measured and if they have been adjudicated or validated in the population of interest	Pg 2,7 Sect 2.5,4	
	92. Provide a data dictionary that includes information on data sources, validity and definitions for all variables, as applicable		NR
	93. Specify definitions and lookback windows for all variables	Pg 3-6 Sect 3	
	94. Report whether any variables could be time-varying (e.g., how the variable could change over time and when it was redefined in relation to time-varying exposures)	Pg 3-6 Sect 3	
	95. Report important variables that could not be captured and their anticipated impact on study results	Pg 3-8 Sect 3,4	
	96. Provide information on deviations from a priori protocol in variable measurements		NA
Section 5: Participants	78. Provide inclusion criteria used to identify study population	Pg 2-3 Sect 2,3	
	79. Justify exclusion criteria and how they may affect the overall interpretation of the research	Pg 3,8 Sect 3,4	
	80. Describe study population characteristics relative to the target population in Canada	Pg 7 Sect 4	

Section	Checklist item	Reported on page number (s)	If not reported or applicable, justify why
	81. Provide all codes and algorithms used to define inclusions and exclusion criteria where possible		NR
	82. Specify the time period (e.g., lookback window) over which inclusion and exclusion criteria were assessed	Pg 2-3<br>Sect 2,3	
	83. Recommendations for specific study designs		NA
	6.1 For cohort studies, provide details leading to the analyzed cohort, including definitions for exposure groups, cohort entry and end dates matching criteria, and censoring/follow-up		NA
	6.2 For prospective cohort studies, describe recruitment processes		NA
	6.3 For case-controlled and case-crossover studies, proved details of case and control ascertainment, the source population for nested studies, sampling methods, and matching criteria		NA
	84. Report the number of participants at each stage of the study and reasons for nonparticipation. Consider illustrating this information using a flow diagram	Pg 3-8<br>Sec 3,4	
	85. Provide characteristics of study participants. If not available for feasible explain why	Pg 2<br>Sect 2.2	
	86. Indicate missing data for each variable of interest		NR
	87. Compare treatment or exposure groups		NA
	88. Specify the number of participants included in each analysis and the analysis strategy (e.g., per-protocol, ITT) and provide details on the number of proportion of subjects excluded from each analysis, and the reasons for exclusion	Pg 3-8<br>Sect 3,4	
Section 6: Exposure definitions and comparators	8. Define the requirements for the exposure definition (e.g./single, multiple, or continuous exposure) and relevant start and stop windows for assessing exposures	Pg 2<br>Sect 2.2	

Section	Checklist items	Reported on page number(s)	If not reported or not applicable. Justify why not
	51. Specify data source(s) from which exposure information was obtained, including validity and any limitations in exposure measurement	Pg 2<br>Sect 2.1,2.2	
	52. Specify the exposure-outcome risk window and discuss how it aligns with the known or anticipated relationship between the exposure and outcome timing	Pg 3-8<br>Sect 3.4	
	53. If no comparator was used, justify why not		NA
	54. Define the comparator group(s) (e.g., active comparator, historical comparator)		NA
	55. Provide justification for the comparator used, including potential implications and study design		NA
	56. Discuss any changes in patterns of us of the exposure and comparator(s) over time and how they may affect the results. Report any methods used to adjust for these changes.		NA
	57. Specify how adaptations to the intervention and/or comparator were permitted and recorded		NA
Section 7: Outcomes	50. Report definitions for all study outcomes (primary, secondary, and exploratory), where possible	Pg 3-6<br>Sect 3,4	
	51. Provide a rationale for the outcomes studied and discuss relevant outcomes not included in the study. Consider the use of a core outcome set if one is available for the condition of interest under study	Pg 3-7<br>Sect 3,4	
	52. Provide information about the validity of all outcome definitions	3-6, 8<br>Sect 3,4	
	53. Describe whether the timing of the outcome can be accurately measured	Pg 8<br>Sect 4	
	54. Specify whether the outcome studied is a surrogate measure of a clinical (patient-centered) outcome and, if so, the strength of the relationship between the surrogate outcome and major clinical outcome(s)of interest	Pg 7<br>Sect 4	
	55. Discuss whether outcome misclassification could occur between treatment groups	8-9	

Section	Checklist item	Reported on page number(s)	If not reported or applicable, justify why not
	56. Report whether a control outcome was used and justify the control outcome(s)selected		NA
Section 8: Bias, confounding, and effect modifiers or subgroup effects	78. Report all procedures used ot address potential sources of bias		NR
	79. Specify how potential sources of bias could influence the outcomes of the analysis		NR
	80. Specify variables that were considered known or potential confounders in the analysis		NR
	81. Describe how confounder variables were selected and if they were informed of a causal diagram		NR
	82. Describe and compare the distribution of measured baseline confounding variables between treatment groups		NR
	83. Report whether any potential confounders could not be measured and specify the anticipated impact of these confounders on study results		NR
	84. Report whether time-varying confounding was considered and if not considered, why not		NR
	85. Specify the methods used to conduct assumptions and limitations of the data and, if no sensitivity analyses were conducted, explain why not		NR
	86. Specify known or potential effect modifiers		NR
	87. Describe any effect modification or subgroup analyses that were conducted and if they were specified a prior. Include if they were identified and conducted based on prespecified rationale. If no effect studies or biological rationale. If no effect modification or subgroup analyses were used, justify why they were not needed		NR
	88. If effect modification or subgroup analyses were used, describe the methods and present separate results for each group		NR
Section 9: Statistical methods	57. Indicate the software used for the statistical analysis, including software package Version, and analytic tools employed (e.g., macros)		NR

Section	Checklist items	Report on page number(s)	If not reported or not applicable, justify why
	58. Provide access to the statistical code used or, if the code cannot be shared, explain why		NR
	59. Report all statistical methods used and justify their selection, including as applicable		
	3.1 all variables included in regression models		NR
	3.2 the method of variable selection for regression models	Pg 3 Sect 3	
	3.3 methods used to control for confounding		NR
	3.4 methods used for accounting for missing data		NR
	3.5 how follow-up time and changes in exposures were handled	Pg 3 Sect 3	
	3.6 subgroup analyses and effect modification	Pg 5 Sect 3	
	3.7 as applicable, stratification, propensity score estimation and assumptions, meta-analysis methods, validity of instrumental variables		NR
	60. Quantify the precision of all estimates using confidence intervals		NR
	61. Report the threshold of the statistical significance used		NR
Section 10: Study findings	36. Summarize key results (estimated effect measures, measures of precision) with reference to each study objective and/or hypothesis for primary and secondary outcomes, and delineate these results by each treatment or exposure group	Pg 3-6 Sect 3,4	
	37. Provide numbers of outcome events or summary measures of outcomes (or exposures in case-control studies)	Pg 3-6 Sect 3	
	38. Report both absolute and relative effect measures for binary outcomes, including their measure of precision		NR
	39. Report category boundaries when continuous variables are categorized and consider translating estimates of relative risk into absolute risk		NR
	40. Report unadjusted and adjusted estimates, including their measure of precision and confounders used for adjustment		NR

Section	Checklist items	Reported on page number (s)	If not reported or not applicable, justify why
	62. Report other prespecified analyses conducted (e.g.,subgroup analyses interactions, sensitivity analyses)		NR
	63. Describe any unplanned analyses performed secondarily (e.g., not defined a priori) and indicate these as exploratory	Pg 8<br>Sect 4	
	64. Avoid selecting reporting of results		NR
Section 11: Interpretation and Generalizability	50. Provide an interpretation of the primary and secondary study results, as applicable	Pg 6-7<br>Sect3,4	
	51. Interpret the findings from adjusted and unadjusted results as applicable		NR
	52. Discuss the Precision of the effect measure(s)	Pg 3, 5-6<br>Sect 3,4	
	53. Discuss how potential biases and sensitivity of study assumptions may impact the results and subsequent interpretation		NR
	54. Discuss the implication of findings of clinical practice, including the risk-benefit profile of the treatment, if applicable	Pg 6-7<br>Sect 3,4	
	55. Interpret study findings in relation to current literature	Pg 6-7<br>Sect 3,4	
	56. Discuss the generalizability (external validity) of study results to the population in Canada	Pg 8-9<br>Sect 4	
Section 12: Limitations	15. Provide consideration of limitations of the study, including the data source, missing data, bias and confounding, imprecision or sample size limitations, and whether results are clinically meaningful	Pg 7<br>Sect 4	
	16. Discuss the plausibility of results and whether results could be due solely to chance, or confounding		NR

Supplemental Table S10: Erenumab in Migraine (20) https://doi.org/10.1111/head.14218

Section	Check item	Reported on page number(s)	If not reported or applicable, justify why
Section 1 : Study design and research questions	89. Report a clearly stated am and study question	79	
	90. Report the overall study design	79,81	
	91. Provide a rationale for the choice of study design	79	
	92. Provide a relevant review of the literature to evaluate pertinent information and knowledge	79, 83-84	
	93. Describe key elements of the study design (e.g., matching)		NA
	94. Consider the use of study diagrams to illustrate key aspects of the study design	81	
	95. Strongly recommend to develop and reference an a priori protocol		NR
	96. Describe all study team members, including the role of patient partners, and any conflicts of interest	88	
	97. Describe the study governance structure, especially who was responsible for final decision-making		NR
	98. Report any research ethics approval (or equivalent)	80	
	99. Disclose sources of funding		NR
Section 2: Setting and Content	33. Describe important information to contextualize the data source, including:		
	1.1 type of care setting	79-80	
	1.2 geographic location	79	
	34. Describe all relevant study period dates, including periods of recruitment, exposure, follow-up, and data collection	79-80	
	35. Clearly identify missing data components in the data collection	80	
	36. For studies that propose the use of a data source from a country other than Canada, Provide		NA

Section	Checklist item	Reported on page number(s)	If not reported or applicable, justify
	4.1 a rationale for selecting the data source		NA
	4.2 an explanation of how these factors might affect the generalizability of the study results to the population in Canada		NA
	4.3 background information about the healthcare system		NA
	4.4 description of prescribing and utilization practices		NA
Section 3: Data specifications- access, cleaning methods, and linkage	81. Describe the extent to which the investigation had access to database population used to create the study population and major aspects of data provenance	80	
	82. Provide information on the data-cleaning methods used in the study. Share any data-cleaning code leveraged. If not, provided, justify	80	
	83. Report whether data were organized by a Common Data Model structure		NA
	84. Describe the usage of data and consent for data sharing. Provide consent documents, if relevant		NA
	85. Describe data collection methods		NA
	86. Quality of the data and relevant metrics to assess the data quality should be reported		NA
	87. Describe any variability between data sources and the impact of changes over time in the data		NA
	88. Describe if any data linkage was conducted and the methods used for the linkage		NA
	89. Report who (e.g., which organization) performed the data linkage, if applicable		NA
	90. Describe the performance characteristics of the data linkage and the number of individuals linked at each stage of linkage		NA

	Checklist item	Reported on page number(s)	If not reported or applicable, justify
Secti0n 4: Data sources, data dictionary and variables	97. Provide and describe all data sources, including the specific version and date of the last update of the database	80	
	98. Describe the characteristics of the health setting and context of data collection	79-80	
	99. Describe details of data continuity and completeness	80	
	100. Include the names, dates, and/or version numbers of when data were extracted for research use; by the data vendor or organization		NA
	101. Include the search and/or extraction criteria applied if the source data are a subset of the data from the vendor or organization, and provide calendar data ranges		NA
	102. Provide source(s)of data for each variable of interest	80	
	103. Describe how variables of interest were measured and if they have been adjudicated or validated in the population of interest	80-81	
	104. Provide a data dictionary that includes information on data sources, validity and definitions for all variables, as applicable		NR
	105. Specify definitions and lookback windows for all variables	80	
	106. Report whether any variables could be time-varying (e.g., how the variable could change over time and when it was redefined in relation to time-varying exposures)		NR
	107. Report important variables that could not be captured and their anticipated impact on study results	87	
	108. Provide information on deviations from a priori protocol in variable measurements	80	
Section 5: Participants	89. Provide inclusion criteria used to identify the study population	80	

	90. Justify exclusion criteria and how they may affect the overall interpretation of the research	80	
	91. Describe study population characteristics relative to the target population in Canada		NR
Section	Checklist item	Reported on page number (s)	If not reported or applicable, justify why
	92. Provide all codes and algorithms used to define inclusions and exclusion criteria where possible		NR
	93. Specify the time period (e.g., lookback window) over which inclusion and exclusion criteria were assessed	80-81	
	94. Recommendations for specific study designs		
	6.1 For cohort studies, provide details leading to the analyzed cohort, including definitions for exposure groups, cohort entry and end dates matching criteria, and censoring/follow-up	79-81	
	6.2 For prospective cohort studies, describe recruitment processes		
	6.3 For case-controlled and case-crossover studies, proved details of case and control ascertainment, the source population for nested studies, sampling methods, and matching criteria		
	95. Report the number of participants at each stage of the study and reasons for nonparticipation. Consider illustrating this information using a flow diagram	81	
	96. Provide characteristics of study participants. If not available for feasible explain why	80-81	
	97. Indicate missing data for each variable of interest	80	
	98. Compare treatment or exposure groups		NA
	99. Specify the number of participants included in each analysis and the analysis strategy (e.g., per-protocol, ITT) and provide details on the number of proportion of subjects excluded from each analysis, and the reasons for exclusion		NA
Section 6: Exposure	9. Define the requirements for the exposure definition (e.g./single, multiple, or continuous	80-81	

definitions and comparators	exposure) and relevant start and stop windows for assessing exposures		
Section	Checklist items	Reported on page number(s)	If not reported or not applicable. Justify why not
	58. Specify data source(s) from which exposure information was obtained, including validity and any limitations in exposure measurement	80	
	59. Specify the exposure-outcome risk window and discuss how it aligns with the known or anticipated relationship between the exposure and outcome timing	81-83	
	60. If no comparator was used, justify why not		NA
	61. Define the comparator group(s) (e.g., active comparator, historical comparator)		NA
	62. Provide justification for the comparator used, including potential implications and study design		NA
	63. Discuss any changes in patterns of us of the exposure and comparator(s) over time and how they may affect the results. Report any methods used to adjust for these changes.		NA
	64. Specify how adaptations to the intervention and/or comparator were permitted and recorded		NA
Section 7: Outcomes	57. Report definitions for all study outcomes (primary, secondary, and exploratory), where possible	81-83	
	58. Provide a rationale for the outcomes studied and discuss relevant outcomes not included in the study. Consider the use of a core outcome set if one is available for the condition of interest under study	81-83	
	59. Provide information about the validity of all outcome definitions		NR
	60. Describe whether the timing of the outcome can be accurately measured	81-82	
	61. Specify whether the outcome studied is a surrogate measure of a clinical (patient-centered) outcome and, if so, the strength of the relationship between the surrogate outcome and major clinical outcome(s)of interest	83-87	

	62. Discuss whether outcome misclassification could occur between treatment groups	86-88	
Section	Checklist item	Reported on page number(s)	If not reported or applicable, justify why not
	63. Report whether a control outcome was used and justify the control outcome(s)selected		NA
Section 8: Bias, confounding, and effect modifiers or subgroup effects	89. Report all procedures used to address potential sources of bias		NR
	90. Specify how potential sources of bias could influence the outcomes of the analysis	87	
	91. Specify variables that were considered known or potential confounders in the analysis		NR
	92. Describe how confounder variables were selected and if they were informed of a causal diagram		NR
	93. Describe and compare the distribution of measured baseline confounding variables between treatment groups		NR
	94. Report whether any potential confounders could not be measured and specify the anticipated impact of these confounders on study results		NR
	95. Report whether time-varying confounding was considered and if not considered, why not		NR
	96. Specify the methods used to conduct assumptions and limitations of the data and, if no sensitivity analyses were conducted, explain why not		NR
	97. Specify known or potential effect modifiers		NR
	98. Describe any effect modification or subgroup analyses that were conducted and if they were specified a prior. Include if they were identified and conducted based on prespecified rationale. If no effect studies or biological rationale. If no effect modification or subgroup analyses were used, justify why they were not needed		NR
	99. If effect modification or subgroup analyses were used, describe the methods and present separate results for each group		NR
Section 9: Statistical methods	65. Indicate the software used for the statistical analysis, including software package Version, and analytic tools employed (e.g., macros)	81	

Section	Checklist items	Report on page number(s)	If not reported or not applicable, justify why
	66. Provide access to the statistical code used or, if the code cannot be shared, explain why		NR
	67. Report all statistical methods used and justify their selection, including as applicable		
	3.1 all variables included in regression models		NR
	3.2 the method of variable selection for regression models	569	
	3.3 methods used to control for confounding		NR
	3.4 methods used for accounting for missing data		NR
	3.5 how follow-up time and changes in exposures were handled		NR
	3.6 subgroup analyses and effect modification		NR
	3.7 as applicable, stratification, propensity score estimation and assumptions, meta-analysis methods, validity of instrumental variables		NR
	68. Quantify the precision of all estimates using confidence intervals		NR
	69. Report the threshold of the statistical significance used		NR
Section 10: Study findings	41. Summarize key results (estimated effect measures, measures of precision) with reference to each study objective and/or hypothesis for primary and secondary outcomes, and delineate these results by each treatment or exposure group	81-86	
	42. Provide numbers of outcome events or summary measures of outcomes (or exposures in case-control studies)	82-87	
	43. Report both absolute and relative effect measures for binary outcomes, including their measure of precision		NR
	44. Report category boundaries when continuous variables are categorized and consider translating estimates of relative risk into absolute risk		NR
	45. Report unadjusted and adjusted estimates, including their measure of precision and confounders used for adjustment		NR

Section	Checklist items	Reported on page number (s)	If not reported or not applicable, justify why
	70. Report other prespecified analyses conducted (e.g.,subgroup analyses interactions, sensitivity analyses)		NR
	71. Describe any unplanned analyses performed secondarily (e.g., not defined a priori) and indicate these as exploratory		NR
	72. Avoid selecting reporting of results		NR
Section 11: Interpretation and Generalizability	57. Provide an interpretation of the primary and secondary study results, as applicable	83-88	
	58. Interpret the findings from adjusted and unadjusted results as applicable		NR
	59. Discuss the Precision of the effect measure(s)		NR
	60. Discuss how potential biases and sensitivity of study assumptions may impact the results and subsequent interpretation		NR
	61. Discuss the implication of findings of clinical practice, including the risk-benefit profile of the treatment, if applicable	83-88	
	62. Interpret study findings in relation to current literature	86-88	
	63. Discuss the generalizability (external validity) of study results to the population in Canada	87	
Section 12: Limitations	17. Provide consideration of limitations of the study, including the data source, missing data, bias and confounding, imprecision or sample size limitations, and whether results are clinically meaningful	87	
	18. Discuss the plausibility of results and whether results could be due solely to chance, or confounding		NR
